# SIRT1 pharmacological activation rescues vascular dysfunction and prevents thrombosis in MTHFR deficiency

**DOI:** 10.1007/s00018-022-04429-5

**Published:** 2022-07-11

**Authors:** Albino Carrizzo, Concetta Iside, Angela Nebbioso, Vincenzo Carafa, Antonio Damato, Sebastiano Sciarretta, Giacomo Frati, Flavio Di Nonno, Valentina Valenti, Michele Ciccarelli, Eleonora Venturini, Mariarosaria Scioli, Paola Di Pietro, Tommaso Bucci, Valentina Giudice, Marianna Storto, Bianca Serio, Annibale Alessandro Puca, Giuseppe Giugliano, Valentina Trimarco, Raffaele Izzo, Bruno Trimarco, Carmine Selleri, Lucia Altucci, Carmine Vecchione

**Affiliations:** 1 Department of Medicine, Surgery and Dentistry, “Scuola Medica Salernitana” University of Salerno, Via S. Allende, 84081 Baronissi, Italy; 2 IRCCS Neuromed, Vascular Physiopathology Unit, 86077 Pozzilli, Italy; 3 IRCCS Synlab SDN, Via E. Gianturco 113, 80143 Naples, Italy; 4 Department of Precision Medicine, University of Campania “Luigi Vanvitelli”, Vico L. De Crecchio, 80138 Naples, Italy; 5 Department of Medico-Surgical Sciences and Biotechnologies, Sapienza University of Rome, 04100 Latina, Italy; 6 Department of Cardiology, Santa Maria Goretti Hospital, 04100 Latina, Italy; 7 University Hospital, San Giovanni di Dio e Ruggi D’Aragona, 84125 Salerno, Italy; 8 IRCCS Multimedica, Ageing Unit, Via G. Fantoli 16/15, 20138 Milan, Italy; 9 Department of Advanced Biomedical Sciences, “Federico II” University, Via Pansini 5, 80131 Naples, Italy; 10 Department of Neuroscience, Reproductive Sciences and Dentistry, “Federico II” University, Via Pansini 5, 80131 Naples, Italy; 11 International Translational Research and Medical Education (ITME) Consortium and Department of Advanced Biomedical Sciences, “Federico II” University, 80131 Naples, Italy; 12 BIOGEM, Institute of Molecular Biology and Genetics, Via Camporeale, 83031 Ariano Irpino, Italy

**Keywords:** MTHFR, SIRT1, Vascular function, Endothelium, Nitric oxide

## Abstract

Beyond well-assessed risk factors, cardiovascular events could be also associated with the presence of epigenetic and genetic alterations, such as the methylenetetrahydrofolate-reductase (*MTHFR*) C677T polymorphism. This gene variant is related to increased circulating levels of homocysteine (Hcy) and cardiovascular risk. However, heterozygous carriers have an augmented risk of cardiovascular accidents independently from normal Hcy levels, suggesting the presence of additional deregulated processes in *MTHFR* C677T carriers. Here, we hypothesize that targeting Sirtuin 1 (SIRT1) could be an alternative mechanism to control the cardiovascular risk associated to *MTHFR* deficiency condition. Flow Mediated Dilatation (FMD) and light transmission aggregometry assay were performed in subjects carrying *MTHFR* C677T allele after administration of resveratrol, the most powerful natural clinical usable compound that owns SIRT1 activating properties. *MTHFR* C677T carriers with normal Hcy levels revealed endothelial dysfunction and enhanced platelet aggregation associated with SIRT1 downregulation. SIRT1 activity stimulation by resveratrol intake was able to override these abnormalities without affecting Hcy levels. Impaired endothelial function, bleeding time, and wire-induced thrombus formation were rescued in a heterozygous *Mthfr*-deficient (*Mthfr*^*+/–*^) mouse model after resveratrol treatment. Using a cell-based high-throughput multiplexed screening (HTS) assay, a novel selective synthetic SIRT1 activator, namely ISIDE11, was identified. Ex vivo and in vivo treatment of *Mthfr*^*+/–*^ mice with ISIDE11 rescues endothelial vasorelaxation and reduces wire-induced thrombus formation, effects that were abolished by SIRT1 inhibitor. Moreover, platelets from *MTHFR* C677T allele carriers treated with ISIDE11 showed normalization of their typical hyper-reactivity. These results candidate SIRT1 activation as a new therapeutic strategy to contain cardio and cerebrovascular events in *MTHFR* carriers.

**Supplementary Information:**

The online version contains supplementary material available at 10.1007/s00018-022-04429-5.

## Introduction

Cardiovascular risk factors, such as smoke and high blood pressure, are the main responsible of cardiovascular morbidity and mortality; however, other conditions, like the occurrence of epigenetic changes and the presence of certain polymorphisms, might be considered not only passengers, while co-drivers for cardiovascular disease development [1]. For example, the methylenetetrahydrofolate-reductase (MTHFR) is an enzyme that plays a central role in remethylation cycle, and catalyzes 5,10-methylenetetrahydrofolate reduction to 5-methyltetrahydrofolate, the principal methyl donor for remethylation of homocysteine (Hcy) to methionine [2, 3]. Impairment in MTHFR activity due to the occurrence of genetic polymorphisms, such as the C677T (heterozygous CT) genotype, can lower the affinity with its cofactors and lead to Hcy accumulation, a well-recognized risk factor of cardiovascular disease through endothelial damage induction and platelet stimulation, adhesion and hyperaggregation [4, 5]. However, the association between *MTHFR* CT genotype and arterial and venous thrombotic diseases as well as myocardial infarction (MI), ischemic stroke (IS), and peripheral vascular disease (PVD), is still under debate because of contrasting results [6–9], and because folate and vitamin B12 administration-related Hcy decrease does not significantly reduce major vascular event recurrence [10, 11]. Therefore, additional deregulated processes are involved in increased cardiovascular morbidity and mortality in the setting of *MTHFR* polymorphisms.

*Mthfr*^*+/–*^ mouse models are characterized by an endothelium-dependent relaxation impairment [12] due to endothelial nitric oxide synthase (eNOS) uncoupling affecting nitric oxide (NO) production [13]. NO, the smallest inorganic gas molecule with endothelial vasodilator abilities, is also produced by platelets [14] contributing to regulation of platelet adhesion, recruitment, and aggregation. NO imbalance is associated with arterial thrombosis and endothelial injury, two possible mediators of poor cardiovascular prognosis in *MTHFR* CT allele carriers. Moreover, *Mthfr*^*+/–*^ mice display an increase in endothelial progenitor cell (EPC) pathological senescence related to NAD-dependent deacetylase sirtuin-1 (SIRT1) downregulation, likely because a synergistic interaction of SIRT1 to for the maintenance of endothelial integrity [13, 15]. However, to date, there is no evidence showing the involvement of SIRT1 signaling in cardiovascular abnormalities in *MTHFR* CT carriers.

The present study was aimed at investigating the role of SIRT1 in reducing endothelial dysfunction and platelet aggregation in *MTHFR* CT subjects and in *Mthfr*^*+/–*^ mice through the administration of pure trans-resveratrol (RSV), a SIRT1 activator by mimicking caloric restriction [16]. Furthermore, we performed a high-throughput multiplexed screening to identify a more selective SIRT1 activator to better define the role of SIRT1 in the pathogenesis of cardio and cerebrovascular events in *MTHFR* CT carriers and to identify a possible therapeutic target for prevention of thromboembolic events.

## Methods

### Reagents

ChemBridge Corporation selected a molecular library of 10,000 commercial compounds (ChemBridge Corporation, San Diego, CA, USA) and were dissolved in DMSO (Sigma-Aldrich, Milan, Italy). EX527 was from Sigma-Aldrich and used at a final concentration of 5 µM; resveratrol (RSV) and etoposide were from Sigma-Aldrich, (Milan, Italy).

### SIRT-GST and NMase-His purification

SIRT1-GST and Nmase-His tag enzymes were purified by *E. coli* BL21 bacteria as previously reported [17].

### SIRT1 fluorescence assay

This assay correlates SIRT1 deacetylase activity with production (and quantification) of ammonia, by coupling two reactions catalized by SIRT1 and nicotinamidase (NMase). In the first reaction, SIRT1 removes the acetyl group from the lysine in position 382 of the peptide p53 (aa 374–389), by reaction with its cofactor NAD + which is cleaved forming O-acetyl-ADP-ribose and nicotinamide (NAM). In the second reaction, the NMase enzyme converts NAM in nicotinic acid and free ammonia (NH3). The assay was performed in 96-well microtiter plate reader with fluorescent readout (Corning 96 Flat Bottom Black Polystyrol). The reaction volume was 25 μL. The reaction buffer was composed by PBS and 1 mM DTT. All compounds of the library, also inhibitor and activator known (used as reference compounds), were dissolved in DMSO and they were tested at 10 μM concentration. SIRT1-purified enzyme (5 μL) of a dilution of 1 mg/mL, was incubated for 15 min at 37 °C with 5 μL of intermediated dilution (50 μM) of compounds or 5 μL of reaction buffer with 0.6% of DMSO for positive control. Then, a mix composed by 5 μL of NMase-purified enzyme, 5 μL of β-NAD intermediated dilution (1 mM) and 5 μL acetylated peptide p53K382 intermediated dilution (250 μM) (synthesized by INBIOS) was added and all mix was incubated for 40 min at 37 °C. Subsequently, developer buffer was added (70% PBS, 30% ethanol, 10 mM DTT and 10 mM OPT (Acros)) followed by a re-incubation for 30 min at RT. Fluorescent signal detection was performed with a microplate reader INFINITE M200/TECAN at 420/460 nm.

### SIRT1 fluorescence assay in HTS mode

A library of 10,000 natural and synthetic molecules was screened by SIRT1 enzymatic assay in high-throughput screening (HTS) mode to identify potential SIRT1 modulators. The primary screening test was carried out using the Tecan Robot Freedom EVO 150 (Tecan Italia Srl, Milan, Italy). The standard assay (SIRT1 Fluorescence assay) was miniaturized in 384 plates (Corning 384 Flat Bottom Black Polystyrene) with a final reaction volume of 15 μL. 384-well plate was previously loaded with the references in the upper left and bottom right-side wells with HP D300 Digital Dispenser TECAN and for each plate 160 compounds were tested in duplicate. On the robot work table, the reaction buffer was placed (PBS, DTT 1 mM and 0.6% DMSO, compounds at 10 mM, SIRT1 and Nmase enzyme mix, which will be used for assay). First, the robot made compounds dilutions (from 10 mM to 10 μM) and it added the mixture of the SIRT1 enzyme to the whole plate except for the negative control wells. After incubation for 15 min at 37 °C, the NMase enzyme mix was added and incubated for 40 min at 37 °C. After that the developer buffer was added for 30 min at room temperature, fluorescence reading was performed by the Tecan INFINITE M1000/Reader. HTS modality yielded highly reproducible data. A SIRT1 activator was defined by an activity ≥ 120% and a SIRT1 inhibitor by an activity ≤ 70%.

### Counter-screening for NMase

To exclude potential activity on NMase enzyme, the active compounds were tested by fluorescence assay in the presence of NMase enzyme only, as previously reported [17]. The assay was carried out in a 96-well plate with the same positive and negative reaction controls of SIRT1 fluorescence assay. Each compound was always tested at 10 μM and so 5 μL of compound intermediated dilution (50 μM) was added to 5 μL reaction buffer. After 40 min incubation at 37 °C, the developer buffer was added and the plate was again incubated in the dark for 30 min at room temperature. Fluorescence reading occurred in the 420/460 nm and it was performed by INFINITE M1000/Reader.

### Evaluation of intrinsic fluorescence

Intrinsic fluorescence at 420/460 nm of the compounds dissolved in the reaction buffer for 40 min at 37 °C was read by an Infinite M1000 reader (Tecan, Italy).

### AC_50_/IC_50_ evaluation

Fluorescence assay was performed in a 96-well plate with each compound loaded from 0.01 to 100 μM using an HP D300 Digital Dispenser (Tecan, Italy). IC_50_/AC_50_ values were obtained using Graphpad Prism software.

### Fluorescence quenching measurements

Fluorescence measurements were obtained using a Perkin Elmer Life Sciences LS 55 spectrofluorometer (Perkin Elmer Life Sciences, MA, USA). Tyrosine fluorescence emission (λ_ex_ 275 nm/λ_em_ 305 nm) was calculated for both full-length SIRT1 (after thrombin cleavage to remove GST) and free tyrosine after addition of ISIDE11 at different doses (0, 0.5, 1, 5, 10, 20, 30, 50 µM). Tyrosyne fluorescence quenching was monitored by estimation of the F0/F ratio, considering the fluorescence intensity at 305 nm of the sample before (F0) and after (F) addition of ISIDE11. Working concentrations were 10 µM for both SIRT1 and free tyrosine.

### Cell lines

Human hepatocellular carcinoma cells (HepG2) were obtained from the American Type Culture Collection (ATCC, Manassas, VA, USA; #HB-8065) and cultured in Dulbecco’s Modified Eagle’s Medium (DMEM; Euroclone, Milan, Italy) supplemented with 10% heat-inactivated fetal bovine serum (FBS; Sigma-Aldrich) and antibiotics (100 U/mL penicillin, 100 mg/mL streptomycin, and 250 mg/mL amphotericin-B). The cells were maintained in an incubator at 37 °C and 5% CO_2_ in a fully humidified atmosphere. HCT-116 (ATCC no. CCL-247), HT-29 (ATCC no. HTB-38), MCF7 (ATCC no. HTB-22), A549 (ATCC no. CCL-185), MiaPaCa (ATCC no. CRL-1420), and A2058 (ATCC no. CRL-11147) cells were grown in DMEM (Euroclone) with 10% FBS (Euroclone), 2 mM l-glutamine (Euroclone), and antibiotics (100 U/mL penicillin and 100 mg/mL streptomycin; Euroclone).

MRC-5 cells (ATCC no. CCL-171) were cultured in Eagle’s minimal essential medium (EMEM; Euroclone) supplemented with 10% FBS (Euroclone) and 10 μg/mL gentamicin solution (Euroclone). U937 cells (ATCC no. CRL-1593.2) were grown in RPMI1640 medium (Euroclone) supplemented with heat-inactivated 10% FBS (Sigma-Aldrich) and antibiotics (Euroclone; 100 U/mL penicillin, 100 mg/mL streptomycin, and 250 mg/mL amphotericin-B). Caco-2 cells (ATCC no. HTB-37) were grown in EMEM (Euroclone) supplemented with 10% FBS (Euroclone) and 10 μg/mL gentamicin solution (Euroclone). HUVECs (ATCC, PCS-100-010) were cultured in Vascular Cell Basal Medium (ATCC, PCS-100-030) supplemented with Endothelial Cell Growth Kit (ATCC, PCS-100-041). 

### Permeability assay

Permeability assay was performed in Caco-2 cells as previously reported [17]. Compound ISIDE11 at 100 μg/ml, lucifer yellow (Sigma-Aldrich) at 100 μM, negative control, and propranolol at 100 μg/mL were solubilized in PBS and 0.1% of DMSO. All compounds were tested in three replicate monolayers.

### FACS analysis

FACS analyses were performed as previously reported [17].

### Cell viability

MTT assay: Cell viability was evaluated by 3-(4, 5-dimethylthiazol-2-yl)-2, 5-diphenyltetrazolium bromide (MTT; Sigma-Aldrich) reduction at a concentration of 0.5 mg/mL for 1 h. Cells (2 × 10^4^ cells/well) were planted in a 96-well plate and incubated with ISIDE11 at different concentrations and times. The absorbance at 570 nm was determined by a Tecan Infinite M200 reader. Cell viability values were expressed as percentage of the control (100%). All experiments were performed in triplicate.

Cell count: After incubation with ISIDE11, an aliquot of cells was suspended with trypan blue (Euroclone) at a ratio of 1:1. Unstained (viable) and stained (nonviable) cells were counted using an optical microscope to calculate the percentage of viable cells.

### Cellular thermal shift assay (CETSA)

MCF7 cells were treated for 1 h with compounds, ISIDE11 at 50 µM and an equal amount of DMSO (vehicle), as control. The cells were harvested and washed with PBS. The respective samples were suspended in PBS (1.5 mL), divided into aliquots (100 μL), and heated at different temperatures for 3 min by Thermo Mixer (Eppendorf, Milan, Italy), followed by cooling for 3 min at 4 °C. After incubation, 50 μl RIPA buffer (50 mM Tris–HCl pH 7.4; 1% NP40; 0.5% Na-deoxycholate; 0.1% SDS; 150 mM NaCl; 2 mM EDTA; 50 mM NaF; one tablet of protease/phosphatase inhibitors) were added to the samples and incubated for 15 min. The samples were then centrifuged at 13,000 rpm for 30 min at 4 °C, the supernatant was recovered, and the protein content was determined using a Bradford assay (Bio-Rad). Of the total protein extract, 20 μg was loaded on 10% SDS-PAGE. Nitrocellulose filters were stained with Ponceau Red (Sigma) as an additional control for equal loading. SIRT1 antibody was Abcam. Immunoblotting quantification and statistical analysis: signal intensity quantification of all bands was performed with ImageJ software, and the intensity of the bands was normalized by each treatment to the respective temperature condition of 40 °C, where the intensity of the bands at 40 °C is set to 100%. The graph curve was generated by GraphPad Prism using the interpolating equation 'Sigmoidal, 4PL, X is log(concentration)' and Dissociation-exponential one-step decay. The statistical significance of each temperature condition was compared with the means of the treatment groups by Student's t test, as previously reported [18, 19].

### Protein histone extraction

Treated and untreated cells (10^7^ cells/ml) were harvested and lysed in triton extraction buffer (TEB; PBS containing 0.5% Triton X 100 [*v*/*v*], 2 mM PMSF, 0.02% [*w*/*v*] NaN3) for 10 min at 4 °C, with gentle stirring. After centrifugation at 2000 rpm at 4 °C for 10 min, the pellet was washed in TEB and suspended in 0.2 N HCl overnight at 4 °C on a rolling table. The samples were centrifuged and the supernatant was recovered to determine the protein content using a Bradford assay (Bio-Rad, Milan, Italy).

### Western blotting analysis

Western blotting analysis in human cell lines was performed following the recommendations of antibody suppliers and loading 20 µg of total protein extracts on 10% polyacrylamide gels and 8 μg of histone protein extracts on 15% polyacrylamide gels. Antibodies used were: SIRT1 (Abcam Cambridge, UK), P53K382ac (Abcam), P53K379ac (Abcam), PARP (Abcam), ATR (Abcam), ATMphS1981 (Abcam), P53 (Santa Cruz Biotechnology, Heidelberg, Germany), P21 and H3K56ac (Cell Signaling by Euroclone, Milan, Italy), H3K9/14ac ((Diagenode, Belgium), H3K56ac (Cell Signaling), H2A.XphS15, H3K18ac (Abcam). ERK1/2 (Santa Cruz Biotechnology), tubulin (Sigma-Aldrich), H4 (Abcam), and H3 (Abcam) were used as loading controls. Western blots on tissues were performed as previously described [20]. Briefly, 50 μg of extracted tissues for each sample were separated by SDS-PAGE and transferred onto a nitrocellulose membrane. Blocked membranes were incubated with primary antibodies in TBS-Tween and 5% milk (or 5% BSA for phospho-specific antibodies) overnight. Blocked membranes were then incubated with anti-p-eNOS Ser1177 (1:1000), anti-p-eNOS Thr495 (1:800), anti-total eNOS (1:1000), SIRT1-P^S47^ (1:500), total SIRT1 (1:1000) or anti-Actin (1:2000). For cellular models, 20 µg of total proteins were loaded on a 10% polyacrylamide gel and 8 μg of histone protein extracts on a 15% polyacrylamide gel. Antibodies used were: SIRT1, P53K382ac, P53K379ac, PARP, ATR, ATMphS1981, H3K18ac, H2A.XphS15, H4, and H3 (all from Abcam); P53 and ERK1/2 from Santa Cruz; P21 and H3K56ac (Cell Signaling); H4K16ac, H3K9/14ac (Diagenode); and tubulin (Sigma-Aldrich). ERK1/2 were used as loading controls. Low-temperature SDS-PAGE (LT-PAGE) was performed for detection of SDS-resistant eNOS dimer and monomer, as described previously [20].

### RNA extraction, RT-PCR, and real-time PCR

Total RNA was extracted in Trizol (Invitrogen, Carlsbad, CA, USA). One μg of RNA was reverse-transcribed using SuperScript VILO (Invitrogen) according to the manufacturer’s protocol. mRNA levels of the analyzed genes were measured by RT-PCR amplification using iQ SYBR GREEN Supermix (Bio-Rad Laboratories) according to the manufacturer’s instructions. Relative mRNA quantification was calculated using the comparative ΔΔCT method. The sequences of primers used for PCR were: SIRT1 FW 5′-TATGCTCGCCTTGCTGTAGA-3′, REV 5′-AACCTGTTCCAGCGTGTCTA-3′; P53 FW 5′-TGCATTTCTTTTTCTGGATT-3′, REV 5′-ACGTAACTACGGCACAAAGT-3′; ACTIN FW 5′-CATGTACGTTGCATCCAG-3′, REV 5′-CTCCTTAATGTCACGCAC-3′.

### Flow-mediated dilatation (FMD) of brachial artery

FMD measurements were performed by a single operator trained for this methodology with an intra-observer variability < 5% and following published guidelines [21]. Measurements were performed before and after administration of 99% pure 100 mg trans-resveratrol (resVida) (Epic4Health, Melville, NY 11,747) for 21 days. For image acquisition of the right brachial artery, a 10-MHz linear probe connected to a Hi Vision Preirus ultrasound system (Hitachi Hi Vision Preirus, Hitachi Medical Corporation, Tokyo, Japan) was used. Baseline images were obtained for 2 min, then the right brachial artery was occluded by inflating the cuff to above 250 mmHg, and kept inflated for 5 min. Subsequently, the cuff was deflated, and images were captured. FMD was calculated using the following formula: (maximum diameter-baseline diameter)/baseline diameter) × 100 [22].

### Platelet aggregation measurement

After centrifugation at 1500* g* of whole blood, platelet aggregation was assessed at 37 °C with constant stirring (1200 rpm) in a lumi-aggregometer (PAP-8; Chrono-Log). Maximum aggregation was expressed as the percentage of maximum light transmission using unstimulated PRP as negative (0%) and stimulated PRP as positive (100%) control. Platelet aggregation was measured as the increase in light transmission for 5 min with the addition of 0.8 μg/ml collagen (Biodata) used as pro-aggregatory stimulus.

### Platelet isolation and vascular studies using platelets-derived supernatants

Platelet-rich plasma (PRP) was obtained after centrifugation at 130*g* for 20 min of 25 mL of whole blood collected in acid citrate dextrose (ACD; 85 mmol/L sodium citrate, 65 mmol/L citric acid, and 125 mmol/L dextrose; 2.5 ml ACD:25 mL of blood). Platelets were then pelleted by PRP centrifugation at 900* g* for 7 min and were resuspended in Tyrode’s solution (132 mmol/L NaCl, 4 mmol/L KCl, 1.6 mmol/L CaCl_2_, 0.98 mmol/L MgCl_2_, 23.8 mmol/L NaHCO_3_, 0.36 mmol/L NaH_2_PO_4_, 10 mmol/L glucose, 0.05 mmol/L Ca-Titriplex, and gassed with 95% O_2_, 5% CO_2_ and pH 7.4 at 37 °C). After a further centrifugation step at 900*g* for 4 min, platelets were resuspended in the same solution, allowed to equilibrate for 10 min at 37 °C and then stimulated with insulin (10 mol/L) for 10 min. Some experiments were performed in pre-treated platelets with *N*-nitro-l-arginine methyl ester (L-NAME) (300 mmol/L for 30 min). After stimulation, platelet suspension was centrifuged for 2 min at 900*g*, and increasing doses of supernatant (0.1–0.2–0.4–0.8 mL) were added to phenylephrine-precontracted arteries mounted in an organ chamber (final volume, 15 mL). Total number and purity of platelets for each preparation was assessed by flow cytometry. Total protein from each preparation was also determined.

### Serum nitrite measurement

The measurement of nitrite levels in serum was performed with a 280i Nitric Oxide Analyzer (Sievers) as previously described [23]. This method is specific for nitrite, since the reaction mixture does not release NO from any other NOx metabolites.

### Animal experiments

All procedures were conducted according to ARRIVE guidelines. Male and female heterozygous (+ /^_^) *Mthfr*-deficient mice and littermate wild-type (+ / +) control mice (C57BL/6 N) aged 11 to 13 weeks were studied. RSV (Sigma-Aldrich) was freshly prepared in saline solution with 3% DMSO. ISIDE11 (ChemBridge Corporation, USA) was freshly prepared in sesame oil solution with 3% DMSO. Both compounds were administered chronically and intraperitoneally (i.p) at 10 mg/kg of body weight/day for 21 consecutive days. Control mice (vehicle group) received saline (i.p.) containing 3% DMSO, as previously described [24, 25]. The dose of trans-resveratrol was selected based on earlier reports, showing its antioxidant property with a concentration ranging from 8 to 40 mg/kg [26]. Mice were killed by decapitation before to perform vascular reactivity studies. Blood samples were collected to evaluate the following haematological parameter: erythrocytes, hemoglobin, hematocrit, mean corpuscular volume (MCV), mean corpuscular haemoglobin (MCH), mean corpuscular haemoglobin concentration (MCHC), and total and differentiated analysis of leukocytes and platelets.

### *Mthfr*-genotype determination in mouse model

Mice were obtained from Prof. Rima Rozen, Child Health and Human Development Program, Centre for Translational Biology, Canada. Mice were genotyped by PCR using the following primers: sense primer 1 (5′-GAA GCA GAG GGA AGG AGG CTT CAG-3′) in exon 3, sense primer 2 (5′-AGC CTG AAG AAC GAG ATC AGC AGC-3′) in the *neo*r gene and antisense primer 3 (5′-GAC TAG CTG GCT ATC CTC TCA TCC-3′) in intron 3. Sizes of amplified fragments of wild type and targeted alleles are 145 bp and 216 bp, respectively. PCR conditions were: 94 °C denature 3 min, then 6 cycles 94 °C 30 s, 69 °C 1 min, 30 cycles 94 °C 30 s, 68 °C 1 min and 30 s, 72 °C 5 min.

### Vascular reactivity studies

Murine mesenteric arteries were mounted in a pressure myograph (Mulvany) within a temperature-controlled “organ bath” containing 5 mL of oxygenated Krebs Solution (95% O_2_–5% CO_2_) (pH ∼ 7.40), and then equilibrated in this ex vivo conditions. During this period and during each experiment, pH and temperature of all buffer solutions were checked at 30-min intervals, and organ bath contents were replaced at 15-min intervals. At the end of equilibration, both endothelial and muscular vascular functions were, respectively, tested using acetylcholine (ACh) and nitroglycerin (NG) after a pre-constriction with increasing doses of phenylephrine (from 10^–9^ to 10^–5^ mol/L; Sigma-Aldrich). Endothelial and vascular functions from heterozygous *Mthfr*^*+/–*^ model and *Mthfr* wild type (^+/+^) controls were also tested before and after ex vivo exposure to 25 μM of resveratrol (RSV), and, in parallel, to 50 and 100 μM of ISIDE11, or to a selective eNOS inhibitor (*N*(*ω*)-nitro-l-arginine methyl ester, l-NAME; 300 μM), or in the presence of the endothelium-derived hyperpolarizing factor (EDHF) inhibitors, apamin (APA, 100 nM) and charybdotoxin (CTx, 100 nM), or in the presence of a selective SIRT1 inhibitor, EX-527 (5 μM).

### In vivo thrombosis model

Animals were randomized to receive RSV (10 mg/kg), placebo (saline), or RSV (10 mg/kg) plus EX527 (5 mg/Kg/die), or another group was randomized to receive ISIDE11 (10 mg/kg), placebo (saline), or ISIDE11 (10 mg/kg) plus EX527 (5 mg/Kg/die) for 21 days. Animals were then subjected to wire-induced endothelial damage and vascular thrombosis for 5 h at the right leg, as previously described [27]. After 5 h from endothelial damage, mice were immediately anesthetized with ketamine and xylazine for Doppler ultrasound analysis conducted with a Visualsonic Vevo and a 30-MHz probe for visualization of the femoral artery (FA) of the right leg and measurement of peak blood flow velocity at the middle level, as well as for the left leg, considered as internal control. After analysis, mice were euthanized and FA from the two legs were harvested and stored in OCT at − 20 °C for histological analyses on sequential sections for assessment of thrombus extension. Percentages of vessel lumen area in hematoxylin–eosin staining were calculated to evaluate the effect of RSV or ISIDE11 treatment.

### Statistical analysis

All data shown are presented as means ± SEM. Statistical analysis was performed by two-way ANOVA followed by “Bonferroni’s multiple comparisons test” for all vascular reactivity studies. One-way ANOVA followed by “Tukey’s multiple comparisons test” has been used for western blot analysis. A *P* value < 0.05 was assigned statistical significance.

Student’s *t* test has been used for QRT-PCR analysis.

### Study approval

All human experiments were performed in accordance with the Declaration of Helsinki and after approval of the Ethical Committee of Istituto Neurologico Mediterraneo I.R.C.C.S. Neuromed (Prot.no. 13619). Based on *MTHFR* genotype, subjects had a wild-type CC (*N* = 10; mean age, 46.5 ± 8 years), a heterozygous CT (*N* = 14; mean age, 46.4 ± 11 years) and a homozygous TT (*N* = 13; mean age, 45.5 ± 11 years) genotype. All subjects were age matched. CT and TT carriers were under B12 and folic acid treatment to control Hcy levels. The animal protocol was approved by IRCCS Neuromed (Ethical approval Number 570/2019-PR).

## Results

### Daily RSV intake improves endothelial flow and reduces platelet hyperaggregation in subjects carrying *MTHFR* C677T

**Fig. 1:**
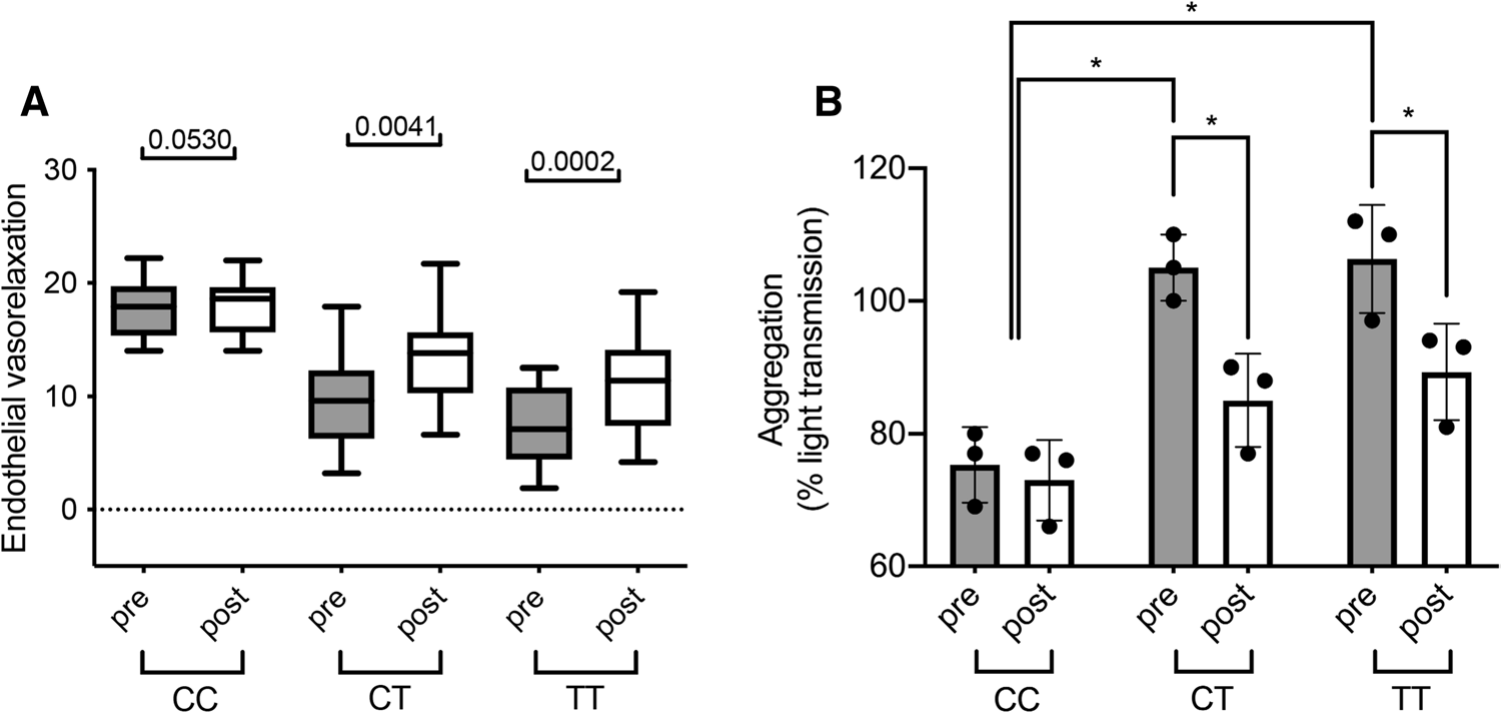
Resveratrol intake improves endothelial function and reduces platelet hyperaggregation in *MTHFR* C677T carriers. **A** Flow-mediated dilation evaluation before and after resveratrol of subjects in our study population divided by genotype (wild type, CC, *N* = 10; heterozygous, CT, *N* = 14; and homozygous, TT, *N* = 13). **B** Quantification of platelet aggregation presented as percentage of light transmission of platelets from control (CTRL) and *MTHFR* carrier subjects. Data are shown are mean ± SEM. *p < 0.05. Statistical analysis was determined by paired two-tailed Student’s t test

CT carrier condition was associated with a marked reduction of endothelial function assessed by non-invasive FMD compared to age-matched CC subjects (Fig. 1A). Interestingly, at 21-days course of RSV (100 mg) SoftGel significantly improved FMD in CT carriers with normal Hcy serum levels induced by previous folate therapy (Fig. 1B). At the end of RSV administration, no Hcy modifications were observed in both genotypes: CT, 12.6±5.1 vs 12.7±2.7 μM, before vs after, respectively; and TT, 12 ± 3.3 vs 12.4±2.7 μM, before *vs* after, respectively. Beneficial RSV action was more pronounced in CT subjects compared to CC carriers (Fig. 1C). We also investigated the effect of RSV treatment on platelet aggregation in humans. Platelets obtained from TT and CT subjects treated with RSV revealed a significant normalization of platelet aggregation in comparison to untreated subjects (Fig. 1D).

### Platelets from CT carriers show reduced NO release

**Fig. 2:**
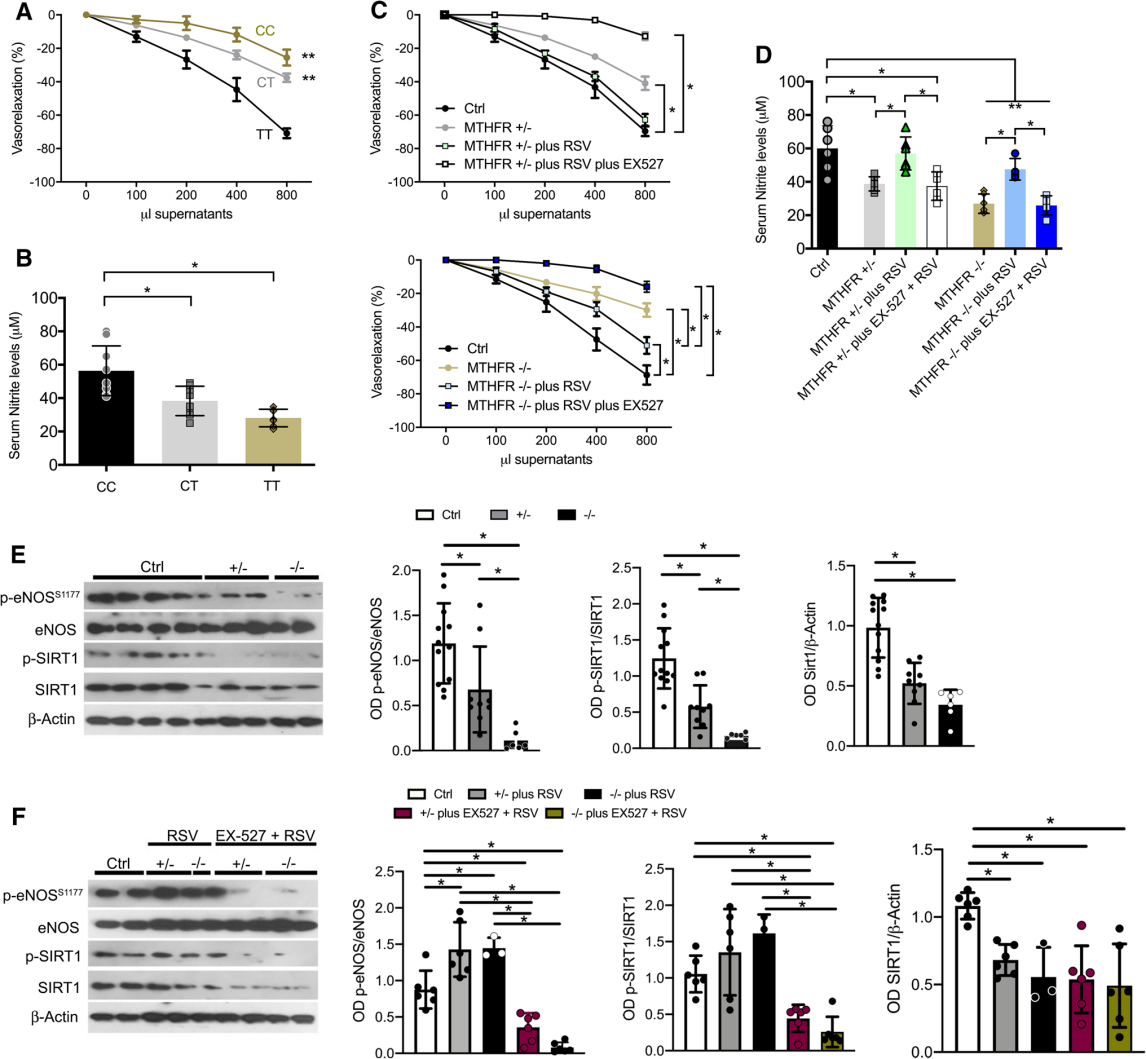
Resveratrol treatment increased NO release, eNOS and SIRT1 phosphorylation in platelets from carriers of the *MTHFR* (C677T) polymorphism. **A** Dose–response curves of phenylephrine-precontracted aorta rings to supernatants derived from insulin-stimulated human platelets isolated from control (Ctrl), *MTHFR* heterozygous (*MTHFR*^*+/–*^), and homozygous (*MTHFR*^–/–^) subjects. **B** Nitrite levels in supernatants derived from insulin-stimulated human platelets isolated from control, *MTHFR*^*+/–*^, and *MTHFR*^–/–^ subjects. (**C**) Dose–response curves of phenylephrine-precontracted aorta rings to platelets’ supernatants obtained from control (Ctrl), *MTHFR*^*+/–*^, and *MTHFR*^–/–^ subjects, treated respectively for 1 h with resveratrol (RSV) or with SIRT1 inhibitor, EX527 (5 uM for 30 min) plus resveratrol. **D** Measurement of nitrite levels in supernatants derived from insulin-stimulated human platelets isolated from control, *MTHFR+/–*, and *MTHFR*–/– subjects with or without treatment with resveratrol (RSV) or EX527 plus resveratrol. **E**, **F** Representative western blots analysis and densitometric analysis of phospho-eNOS on serine 1177, total eNOS, phospho-SIRT1 on serine 47, total SIRT1 and Beta-Actin on platelets obtained from control subjects, *MTHFR*^*+/–*^ and *MTHFR*^–/–^. *p < 0.05. Statistical analysis was determined by two-way ANOVA followed by Bonferroni’s post hoc test

Supernatants from insulin-stimulated human platelets evoked a rapid dose-dependent relaxation of murine aorta rings abolished by l-NAME (data not shown); the vasorelaxant effect was markedly reduced in CT carriers, and even more in TT genotype (Fig. 2A), as also confirmed by significantly reduced supernatant nitrite levels (Fig. 2B). Isolated platelets from heterozygous and homozygous subjects were then treated in vitro with RSV, and platelet-associated vasorelaxant capability significantly improved after RSV treatment but not in platelets pre-treated with EX-527 (Fig. 2C, D). Moreover, NO levels were significantly reduced in CT and TT carriers under insulin stimulation and were associated with decreased eNOS and SIRT1 phosphorylation (Fig. 2D, E), restored after RSV treatment (Fig. 2D and F), whose beneficial actions were lost in the presence of EX-527 (Fig. 2D–F).

### RSV treatment restores endothelial-dependent vasorelaxation throughSIRT1-eNOS axis in vessels of *Mthfr*^*+/–*^ mice

**Fig. 3:**
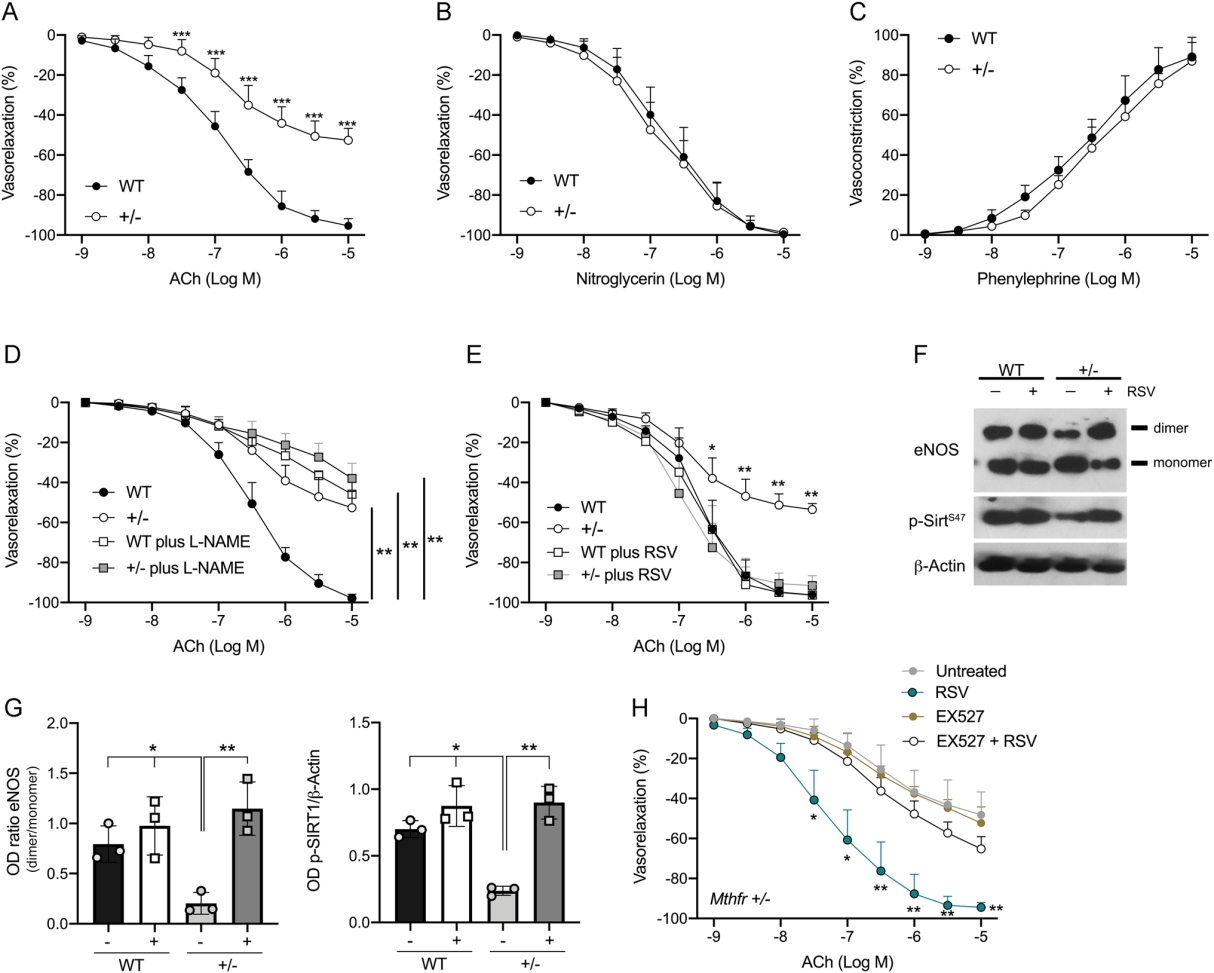
Resveratrol treatment restores endothelial function through SIRT1-eNOS axis in mesenteric arteries from *Mthfr*^*+/–*^ mice. **A**–**C** Graphs show, from left to right, the ex vivo response of mouse mesenteric arteries obtained from wild-type and *Mthfr*^*+/–*^ mice to endothelium-dependent vasorelaxant acetylcholine (ACh), and endothelium-independent vasorelaxant nitroglycerin (NG) and the dose responses to phenylephrine (PE) (*N* = 6 for each experiment). **D** Dose–response curves to acetylcholine (ACh) of mesenteric arteries obtained from *Mthfr*^*+/–*^ mice and their relative littermates in presence of eNOS inhibitor, L-NAME). **E** Dose–response curves to acetylcholine (ACh) after 1 h of resveratrol (RSV) incubation. **F**, **G** Representative western blot analysis of vessels obtained at the end of RSV stimulation and relative quantification. **H** Dose–response curves to ACh after RSV incubation for 1 h both in presence and absence of SIRT1 inhibitor EX527. *p < 0.05. **p < 0.001; ***p < 0.0001. Statistical analysis was determined by two-way ANOVA followed by Bonferroni’s post hoc test

To investigate the possible pathophysiological mechanisms linked to endothelial dysfunction and platelets hyperaggregation we exploit heterozygous *Mthfr* mice model. Assessment of ex vivo vascular function in *Mthfr*^*+/–*^ mice revealed a significant impairment of acetylcholine-evoked vasorelaxation as compared to 10-week-old WT mice (Fig. 3A), in contrast with phenylephrine and nitroglycerin that were unaltered (Fig. 3B, C). The analysis of eNOS phosphorylation at two key regulatory sites of the enzyme [28, 29], revealed significant reduction of the phosphorylation on activation site in serine 1177 in *Mthfr*^*+/–*^ mice, compare to WT counterparts, while no modification on the inhibitory threonine 495 site was detected (Supplementary Fig. 1A). L-NAME-mediated eNOS inhibition significantly reduced endothelial vasorelaxation in WT mice, while in *Mthfr*^*+/–*^ mice there was only a mild, slight reduction of ACh-evoked vasorelaxation (Fig. 3D). To rule out the role of EDHF in our experimental setting, we have performed studies in presence of apamin (APA)—a potent inhibitor of ATP-type Ca^2+^-activated K + channels, and SKCa—and charybdotoxin (CTx)—a potent and selective inhibitor of the voltage-gated Ca^2+^-activated K + channel (Kv1.3) and BKCa channel, widely used to inhibit the endothelium-derived hyperpolarizing factor (EDHF)-mediated pathway. Our studies performed in *Mthfr* deficiency condition, allowed us to exclude the EDHF involvement in ISIDE11 or resveratrol-mediated endothelial vasorelaxation, since the addition of apamin and CTx is able to reduce acetylcholine-mediated vasodilation to the same extent in wild-type and in Mthfr^*+/–*^ mice both in presence of resveratrol or ISIDE11 (Supplementary Fig. 1B–K). In *Mthfr*^*+/–*^ mice, RSV showed, at 25 μM for 1 h, the capability to completely restore endothelial vasorelaxation (Fig. 3E), the dimeric active eNOS form and a concomitant increase of phosphorylated SIRT1 (p-SIRT1) (Fig. 3F and G). Interestingly, EX-527 pretreatment completely abolished RSV-mediated endothelial beneficial effects (Fig. 3H).

**Fig. 4:**
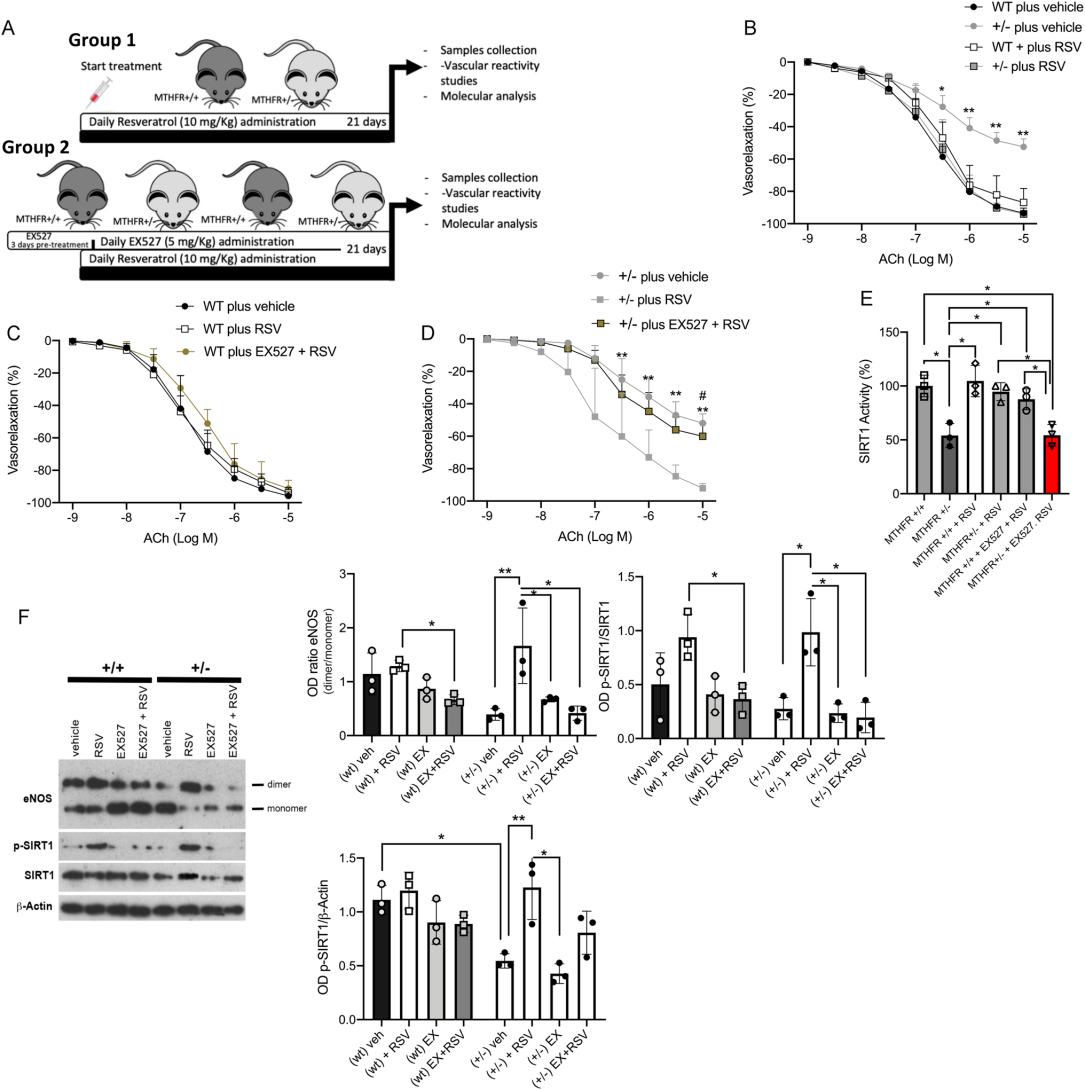
SIRT1 inhibition abolishes eNOS dimerization and endothelial vasodilation evoked by RSV in *Mthfr*^*+/–*^ mice. **A** Schematic representation of in vivo animal treatment. **B** Dose–response curves to acetylcholine (ACh) of mesenteric arteries from wild-type and *Mthfr*^*+/–*^ mice collected at the end of in vivo treatment of Group 1. **C, D** Dose–response curves to acetylcholine (ACh) of mesenteric arteries obtained from **C** wild-type and **D** *Mthfr*^*+/–*^ mice collected at the end of in vivo treatment of Group 2, in presence of SIRT1 inhibitor, EX527. **E** Measurement of SIRT1 activity in mice mesenteric arteries collected at the end of in vivo treatment. **F** Representative western blot analysis performed on mesenteric arteries extracted at the end of in vivo treatment. *p < 0.05; **p < 0.001. Statistical analysis was determined by two-way ANOVA followed by Bonferroni’s post hoc test

To validate the beneficial action of RSV observed ex vitro, we performed in vivo studies administering RSV alone (10 mg/Kg; Group 1) or pretreating mice with EX-527 (5 mg/Kg; Group 2) before RSV for 21 days (Fig. 4A, B). *Mthfr*^±^ mice, basally characterized by endothelial dysfunction, showed, after RSV treatment, a complete rescue of ACh-evoked endothelial vasorelaxation which was lost when mice were pre-treated with EX-527 (Fig. 4C and D). The rescue did not involve the nitroglycerin-evoked smooth-muscle vasorelaxation (data not shown). As expected, SIRT1 activity was restored after chronic RSV treatment and completely abolished at the end of concomitant EX-527 treatment (Fig. 4E). As observed ex vivo, RSV induced in vivo eNOS dimerization in heterozygous *Mthfr*^*+/–*^ mice, and an increase of total and phosphorylated SIRT1 expression, which was abolished in the presence of EX-527 (Fig. 4F).

### In vivo administration of resveratrol improves endothelial vasorelaxation and reduces thrombogenesis in *Mthfr*^*+/–*^ condition

**Fig. 5:**
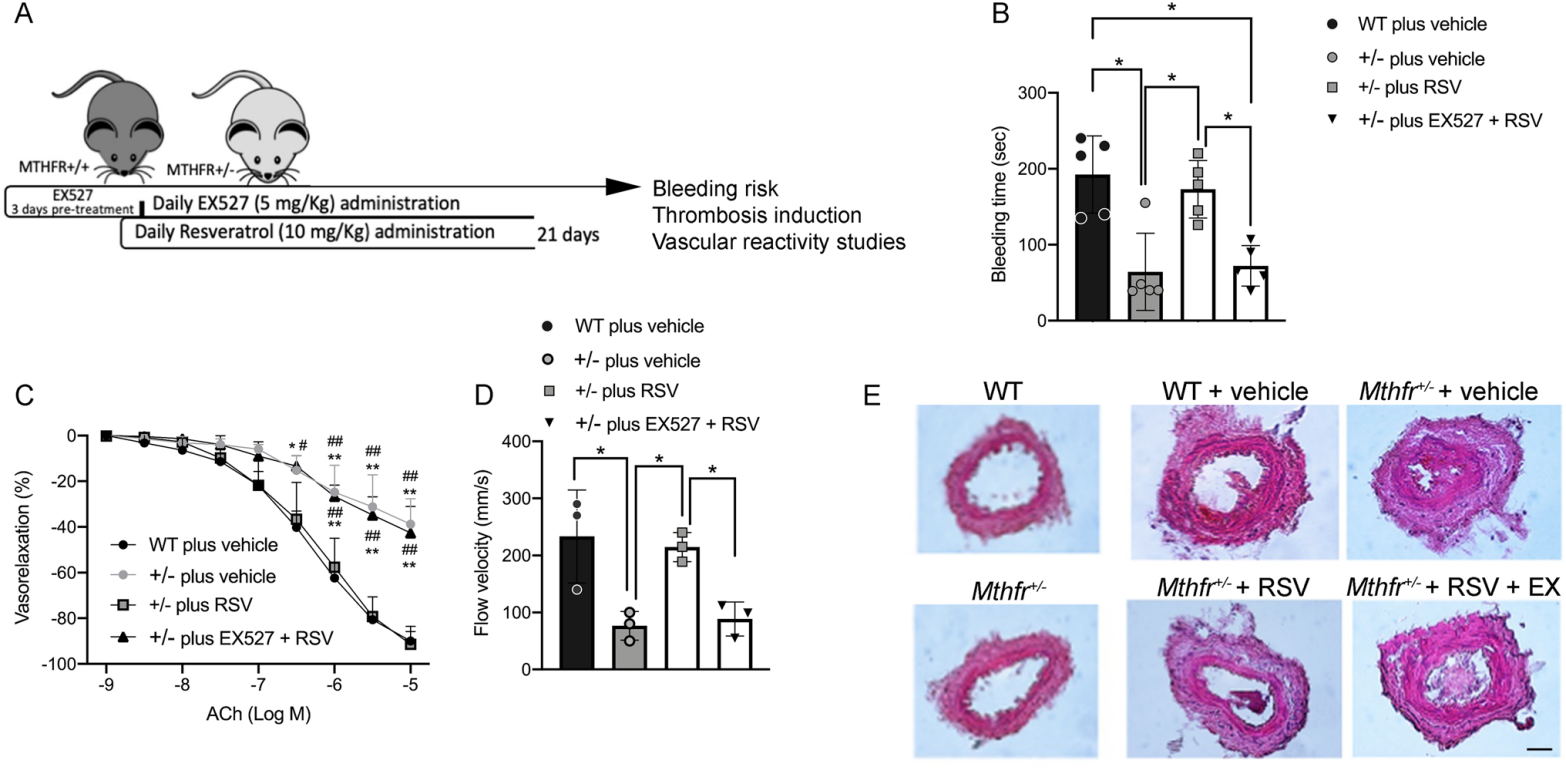
In vivo administration of resveratrol reduces thrombogenesis *in Mthfr*^*+/–*^ mice. **A** Schematic representation of in vivo animal treatment. **B** Tail-vein bleeding times examined in wild-type and in *Mthfr*^*+/–*^ mice in basal condition (vehicle), after 21 days of resveratrol treatment (RSV) and after 21 days of combined treatment with EX527 plus resveratrol (EX527 + RSV). **C** Dose–response curves to acetylcholine (ACh) of mesenteric arteries excised from wild-type and *Mthfr*^*+/–*^ at the end of 21 days of treatment. **D** Blood flow velocity measurement in thrombotic femoral arteries of wild-type and in *Mthfr*^*+/–*^ mice treated as indicated. **E** Representative micro-sections of femoral arteries of mice collected at the end of in vivo treatment after wire-induced endothelial damage and thrombus formation. Images were acquired using a Nikon DS-Ri1 camera with a 20X objective; scale bar, 25 μM. Right graph shows the semi-quantitation of lumen area expressed in percentage. Statistical analysis was determined by two-way ANOVA followed by Bonferroni’s post hoc test. *p < 0.05; **p < 0.001*;* ^#^*p* < 0.05 vs ± plus RSV; ^##^*p* < 0.001 vs ± plus RSV

To assess the potential protective role of SIRT1 activators against thrombosis, we evaluated the bleeding time in wild-type and *Mthfr*^*+/–*^ mice (Fig. 5A). *Mthfr*^*+/–*^ mice showed a significant reduction in bleeding time compared to wild-type (Fig. 5B), which was normalized with RSV treatment; while RSV plus SIRT1 inhibitor EX-527 treatment resulted in a bleeding time similar to that observed in vehicle-treated *Mthfr*^*+/–*^ mice (Fig. 5B), as also confirmed by vascular function assay (Fig. 5C). Moreover, *Mthfr*^*+/–*^ mice showed a significant reduction of peak blood flow velocity after 5 h from induced artery lesion in comparison to WT mice. RSV treatment significantly reduced arterial occlusion and induced normalization of blood flow velocity (Fig. 5D). In addition, immunohistochemical analysis of femoral artery lesions and semiquantitative measurement of the percentage of lumen area revealed a complete prevention from thrombus formation in RSV-treated heterozygous mice, not observed in RSV- and EX527-concomitant-treated mice (Fig. 5E).

### The novel SIRT1 activator ISIDE11 plays a role in endothelial function and thrombogenesis

Given the exciting effects of SIRT1 activation by RSV in rescuing endothelial function and platelet aggregation in MTHFR deficiency in humans and mice, we speculated whether selective SIRT1-targeted activation would be beneficial. Using an in vitro fluorescence-based HTS assay, we screened a library of 10,000 natural and synthetic molecules at 10 μM concentration and identified a new molecule, ISIDE11, as a promising candidate (Supplemental Fig. 2A). ISIDE11 was able to increase the enzymatic activity of SIRT1 by up to about 140% (Supplemental Fig. 2B). The molecule did not modulate NMase activity in counter-screening (Supplemental Fig. 2C). In a concentration–response curve, ISIDE11 displayed an AC_50_ value of 20.11±0.15 μM (Supplemental Fig. 2D).

Given the well-documented direct interaction between SIRT1 and RSV [30, 31], a cellular thermal shift assay (CETSA), a label-free method to assess target binding to compounds in the native cellular environment, was performed in MCF7 breast cancer cells. The signal intensity of the SIRT1 protein detected via Western blot was correlated with temperature to obtain CETSA melting curves. From the derived curves, we calculated the melting temperature, Tm, as the temperature at which 50% of the SIRT1 protein was precipitated. Our results showed that ISIDE11 treatment increased Tm from 46.5 °C (in the control) to 52.05 °C, while RSV treatment increased Tm to 51.4 °C (Supplemental Fig. 2E, bottom), suggesting a stronger binding of ISIDE 11 than RSV. Compared to the control, ISIDE11 protected SIRT1 from the thermal degradation given that SIRT1 signal was still present at the highest temperature (67 °C) in the treated extract, suggesting the direct interaction (Supplemental Fig. 2E, top). Using data processing software, we calculated the dissociation constant (K) of SIRT1-ISIDE11 and SIRT1-RSV binding. The results show a value of 0.015 for ISIDE11 and 0.027 for RSV compared with 0.066 for control. The direct molecular interaction between SIRT1 and ISIDE11 has also been confirmed by fluorescence spectroscopy (Supplemental Fig. 2F). The emission spectra of the fluorescence of several tyrosines of SIRT1 were recorded in the absence and presence of ISIDE11 with different molar ratios. The intensity of the fluorescence decreases regularly as the concentration of ISIDE11 increases, thus indicating that ISIDE11 interacts directly with SIRT1 and induces the decrease in the fluorescence emitted by tyrosine. To exclude a collisional effect by ISIDE11, the same experiment was also performed on the monomeric tyrosine residue (N-acetyl-L-tyrosine ethylester). The F0/F values recorded for free tyrosine were significantly higher than those for SIRT1 at each molar ratio, pointing to the formation of a SIRT1–ISIDE11 complex.

**Fig. 6:**
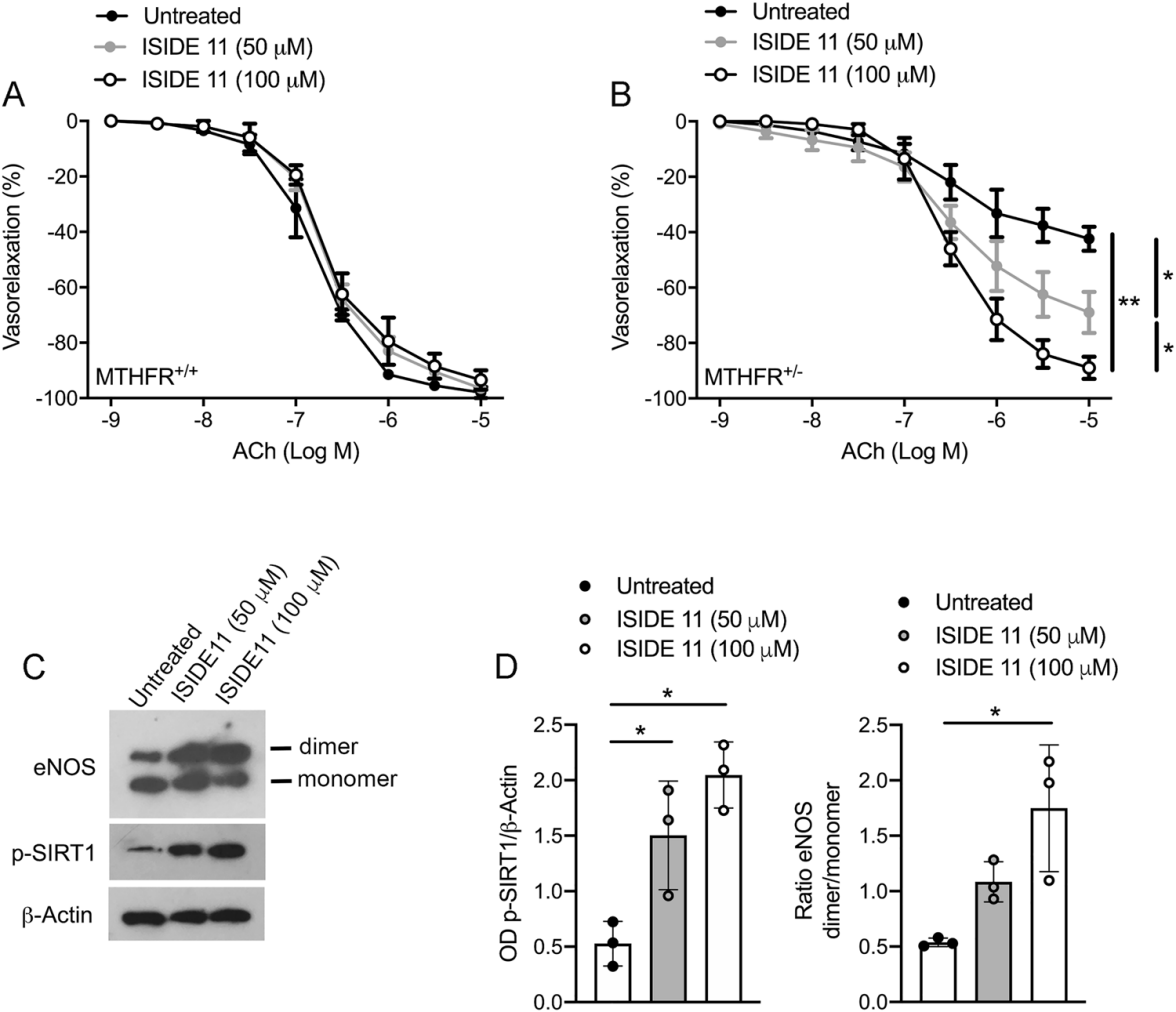
ISIDE11 restores impaired endothelial function in *Mthfr*^*+/–*^ mice. **A**, **B** Dose–response curves to acetylcholine (ACh) of mesenteric arteries obtained from wild-type (**A**) and *Mthfr*^*+/–*^ mice (**B**) in control condition (Untreated), or after treatment with ISIDE11 at the concentration of 50 and 100 uM. **C** Representative western blots analysis and densitometric analysis of eNOS monomer/dimer, phospho-SIRT1 on serine 47, and Beta-Actin in mesenteric arteries from *Mthfr*^*+/–*^ mice. Statistical analysis was determined by two-way ANOVA followed by Bonferroni’s post hoc test. *p < 0.05; **p < 0.001

Since ISIDE11 is able to cross cell membranes (Supplemental Fig. 2G), its effects on cell cycle progression and proliferation were also investigated (Supplemental Fig. 3). In addition, ISIDE11 enhanced SIRT1-mediated effects in stress response pathways, as shown by deacetylation of p53 (Supplemental Fig. 4) upon the strong acetylation induced by reactivity to the chemotherapeutic agent etoposide, supporting its SIRT1 activator action. Interestingly, this effect was stronger than that observed with RSV (Supplemental Fig. 4B). Thus, we explored the ability of ISIDE11 to affect, in vitro, vascular function in wild-type and *Mthfr*^*+/–*^ mice. No changes in ACh-evoked vasorelaxation were observed in wild-type mice (Fig. 6A). Interestingly, a progressive dose-dependent improvement of Ach-evoked vasorelaxation was observed after 1 h of incubation with ISIDE11 already at 50 μM up to a complete restoration at 100 μM (Fig. 6B), indicating the beneficial effect of ISIDE11. In addition, studies performed in presence of EDHF inhibitors, clearly revealed the lack of SKCa and BKCa channels involvement in ISIDE11 vascular actions (Supplementary Fig. 1I-K). Western blot analysis revealed that ISIDE11 was able to positively modulate eNOS dimerization and SIRT1 phosphorylation at Serine 47 (Fig. 6C).

**Fig. 7:**
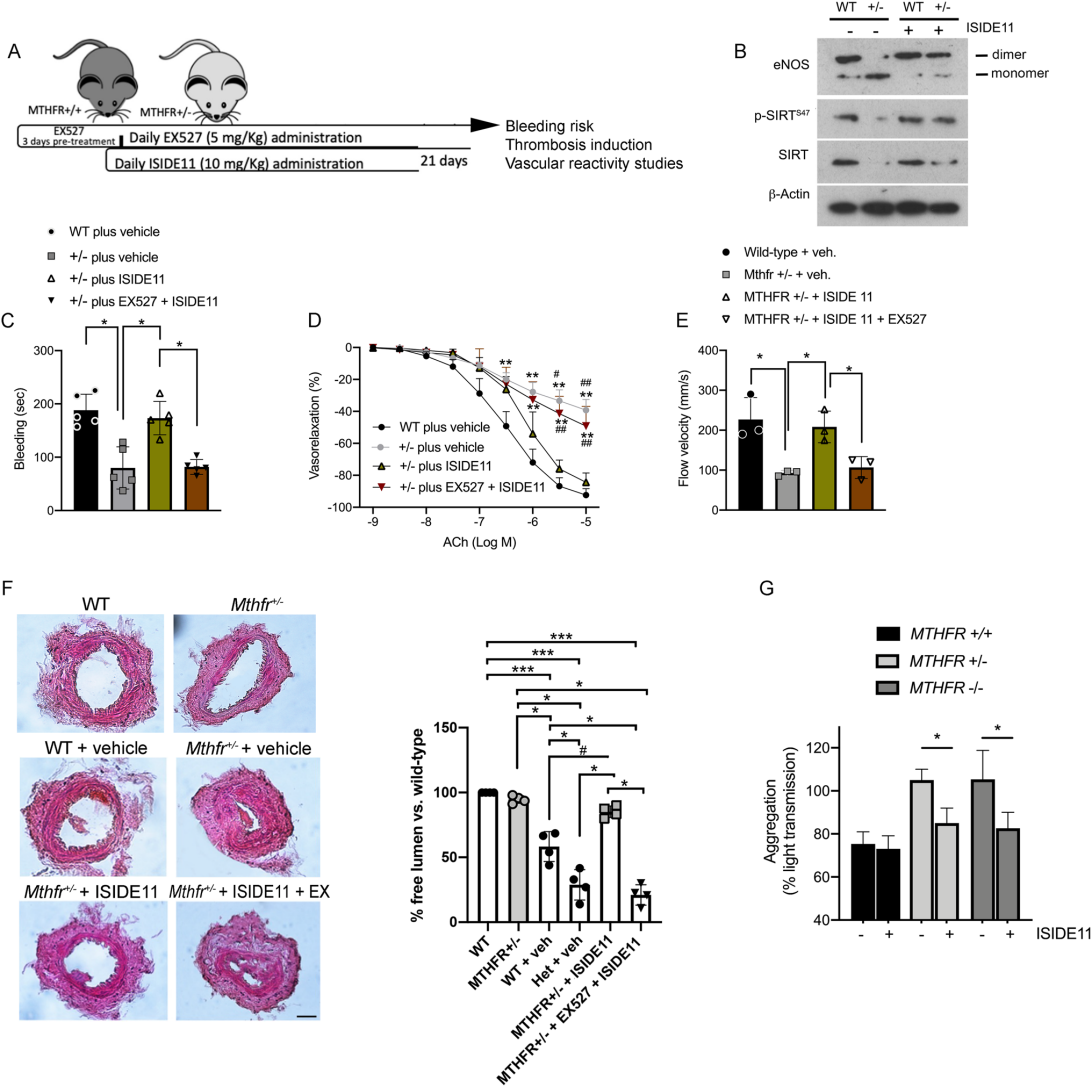
ISIDE11 activates SIRT1 to protect against thrombosis and platelet activation associated with *MTHFR* deficiency. **A** In vivo settings of thrombosis model. **B** Representative western blot analysis of vessels obtained at the end of in vivo treatments. **C** Bleeding time evaluation in *Mthfr* heterozygous mice and relative control littermates treated as indicated. **D** Dose–response curves to acetylcholine (ACh) of phenylephrine-precontracted mesenteric arteries from *Mthfr* heterozygous mice and relative control littermates treated as indicated. **E** Blood flow velocity measurement in femoral artery of *Mthfr* heterozygous mice and relative control littermates treated as indicated. **F** Immunohistochemical analysis of the femoral artery lesion in *Mthfr* heterozygous mice and relative control littermates treated as indicated. Images were acquired using a Nikon DS-Ri1 camera with a 20X objective; scale bar, 25 μM. Right graph shows the semi-quantitation of lumen area expressed in percentage. **G** Aggregation of platelets isolated from WT, homo- and heterozygous *MTHFR* subjects incubated with ISIDE11. Statistical analysis was determined by 2-way ANOVA followed by Bonferroni’s post hoc test. *p < 0.05; **p < 0.001*;* ^#^*p* < 0.05 vs ± plus ISIDE11; ^##^*p* < 0.001 vs ± plus ISIDE11

Similarly, we assessed the potential protective role of ISIDE11 on bleeding time and induced thrombosis in presence or absence of EX-527 concomitant treatment (Fig. 7A). Similarly, to RSV action, treatment with ISIDE11 favored eNOS dimerization, increased SIRT1 phosphorylation and SIRT1 total protein in heterozygous mice (Fig. 7B). A significant reduction in bleeding time was observed in heterozygous mice compared to wild-type mice, an effect that was nullified after ISIDE11 treatment (Fig. 7C). As described for RSV, beneficial effects of ISIDE11 were abolished during the concomitant treatment with SIRT1 inhibitor EX-527. Likewise, endothelial function, peak blood flow velocity, and artery occlusion were significantly improved after ISIDE11 treatment, while EX527 completely inhibited these effects (Fig. 7D–F). The analysis hematological parameters in control and *Mthfr*^*+/–*^ mice groups at baseline and after treatment with ISIDE11 for 21 days did not reveal any statistical change in red and white blood cells features and count. Moreover, the histopathological analyses of livers, spleens and kidneys obtained from different mice groups, revealed no structural alterations compared to organs from wild-type control mice (Supplemental Table 1 and Supplemental Fig. 5). Organs’ structures were well preserved in both control (baseline) and after 21 days of treatment with ISIDE11. In detail, there was no vacuolization in hepatocytes of ISIDE11-treated animals as for absence of leukocyte infiltration around the centrolobular vein, generally detected in presence intracellular lesions. At the spleen level, the structure was well preserved and the lymphatic nodes had similar size between control and ISIDE11-treated animals without expansion regions that are generally correlated with a reduction of lymphocytes number. Finally, the kidneys showed preserved sub capsular space and contorted tubules without alterations between control and ISIDE11-treated animals (Supplemental Fig. 5).

Notwithstanding, since insufficient data are currently available to translate the use of ISIDE11 to clinical trials specifically investigating homo- and heterozygous *MTHFR* C677T subjects, we assessed its potential efficacy on human platelet aggregation. Surprisingly, ISIDE11 was able to significantly reduce hyper-aggregability of platelets isolated from CT and TT carriers, not altering the reactivity of CC carrier-derived platelets (Fig. 7G).

## Discussion

First discovered in 1962 and classified as a rare genetic variant leading to homocystinuria, the *MTHFR* C677T heterozygous genotype is present in 8–20% of the European, Australian, and North American populations, showing a high frequency of this polymorphism. Reduced affinity of MTHFR for its cofactors in CT and TT carriers causes increased levels of Hcy which its involvement in sudden cardiovascular events is still under debate because of contrasting results suggesting only a bystander role of these *MTHFR* polymorphisms [32, 33]. Exercise intervention in a Hcy condition contributes to the reduction of oxidative stress via SIRT1 activation [34]. In contrast, it has been observed that although the levels of Hcy were reduced, this did not promote a reduction in cardiovascular events in humans. Therefore, additional pathological mechanisms not restored by Hcy normalization co-occur in CT and TT carriers who experienced cardiovascular events. Here, we candidate SIRT1 signaling as an important determinant of vascular disease and platelet hyperaggregation in *MTHFR* CT condition. RSV is a natural polyphenolic compound present in various dietary components such as mulberries, peanuts and red wine, and is classified as the most potent SIRT1 activator [16, 35]. In this study, we investigated beneficial effects of RSV-mediated SIRT1 activation using a non-invasive FMD approach in CT and TT carriers responsive to folic acid and vitamin B12 treatment and with normal Hcy levels. FMD assessment clearly showed a significant impairment of endothelial vasorelaxation in CT carriers, and a 21 day-course of pure trans-resveratrol treatment was able to completely rescue endothelial functions. These results suggest that *MTFHR* C677T polymorphism contributes to vascular alteration regardless of circulating Hcy levels, which are not sufficient alone as a prognostic factor for cardiovascular events.

## Conclusions

Here, we documented for the first time the complex crosstalk between MTHFR and SIRT1 signaling pathways in maintenance of cardiovascular homeostasis as supported by RSV-mediated SIRT1 activation and beneficial effects in our *MTHFR* carriers. In our *Mthfr*^*+/–*^ mouse model, RSV administration rescued endothelium-dependent vasorelaxation and normalized bleeding time likely through the restoration of SIRT1 activity. Indeed, SIRT1 inhibition completely suppressed all beneficial cardiovascular effects triggered by RSV, thus demonstrating for the first time the key role of SIRT1 signaling in *Mthfr* deficiency condition. Altered NO bioavailability is a well-known pathogenetic mechanism involved in cardiovascular disease development causing abnormal platelet and coagulation cascade activation [36]. In our study, CT carriers were characterized by an elevated collagen-mediated platelet hyper-reactivity normalized after RSV treatment. Our results suggest that SIRT1 activators could be a novel promising prevention strategy for acute cardiovascular events. RSV has several cardiovascular benefits on ischemic-reperfusion injury [37, 38], platelet functions [39], enhancement of antioxidant status [40], and increase of NO bioavailability [20]. However, long-term RSV administration effects are still under investigation, since some studies have reported few side-effects linked to its long assumption period, such as hepatotoxicity or neutropenia [41–43]. For these reasons, we screened for a more selective SIRT1 activator by an in vitro fluorescence-based HTS assay, and a novel compound, namely ISIDE11, was identified after a high-throughput multiplexed screening of a natural and synthetic library of 10,000 sirtuin-activating compounds (STACs) [44–46]. This small molecule was able to increase SIRT1 activity of 140% by direct binding, and displayed an AC_50_ value of 20.11±0.15 μM. Moreover, our studies showed that ISIDE11 was effective in in vitro and in vivo endothelial-dependent vasorelaxation improvement in dysfunctional vessels from heterozygous *MTHFR* mice. In addition, by reproducing thrombotic events in this mouse model, we revealed the ability of ISIDE11 treatment to limit thrombus formation by restoring normal blood flow in damaged femoral artery through SIRT1 activation. Furthermore, ISIDE11 administration for 21 days did not evoke any toxic effect as shown by hematological parameters and histopathological analysis of liver, spleen and kidneys.

Since ISIDE11 cannot yet be administered in humans due to the lack of clinical trials, we evaluated its action in vitro on hyperaggregation of platelets obtained from *MTHFR* C677T subjects. Our results show that ISIDE11 restores platelet function and NO release, reinforcing the hypothesis of a role for SIRT1 in the pathogenesis of *MTHFR* C677T vascular dysfunction and prothrombotic-associated phenotype.

**Fig. 8:**
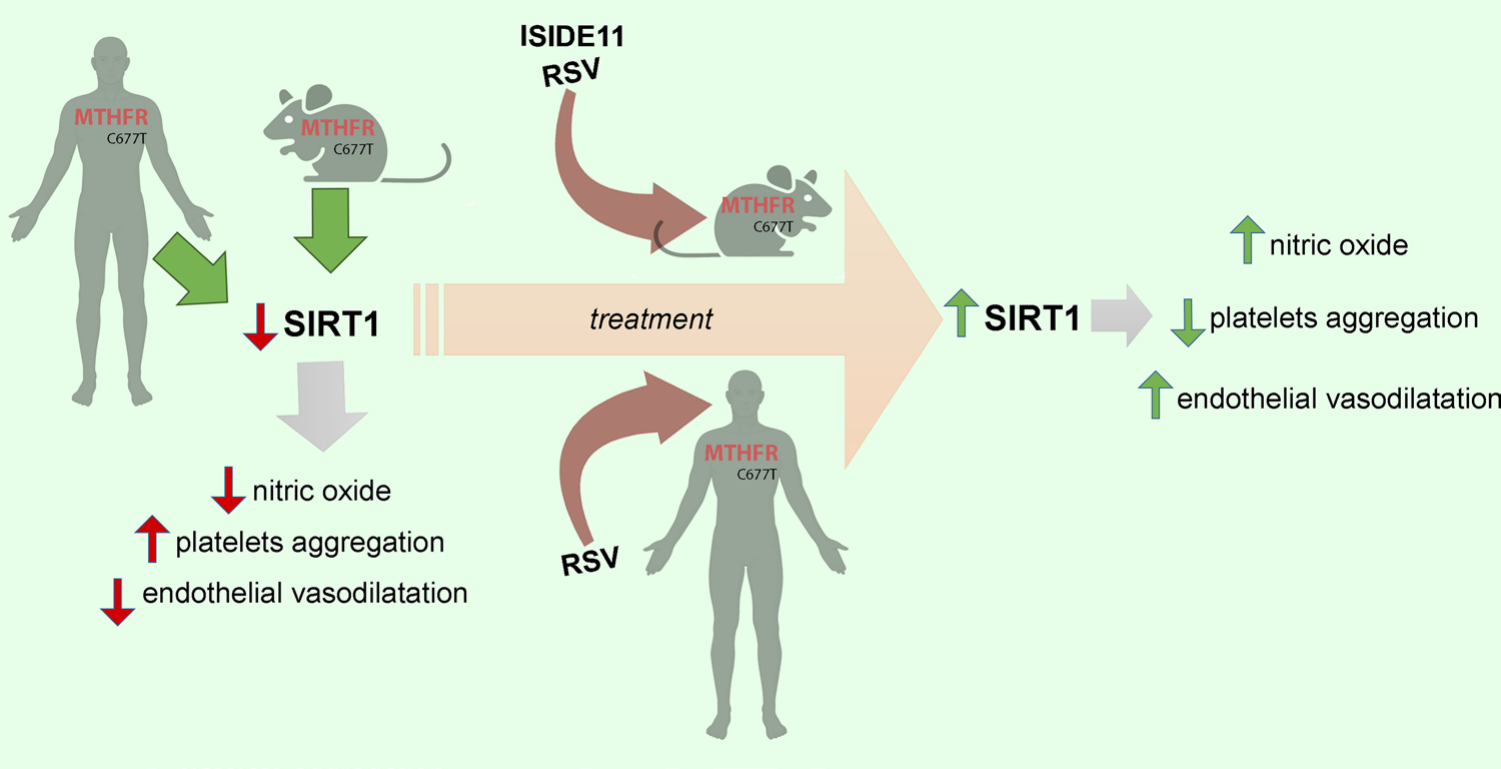
Graphical abstract representing on the left side the steady state of MTHFR C677T carrier subjects and heterozygous mice model characterized by the downregulation of SIRT1 and related impairment of nitric oxide, platelets aggregation and endothelial dysfunction. Central and right side of the image represent the impact of the treatment with resveratrol or ISIDE11 both in mice and human on the improvement of SIRT1 activity that leads to the rescue of nitric oxide production, normalize platelets hyper-reactivity and restore the impaired endothelial function

These findings candidate SIRT1 activation as a new therapeutic strategy to reduce cardio and cerebrovascular events in *MTHFR* C677T carriers (Fig. 8—Graphical abstract). Limitations of our study are that some experiments were conducted on a small number of samples and, because of the limited sample availability, platelet aggregation experiments were the only assay performed to show increased thrombogenicity, while additional methods, such as integrin activation, with multiple agonists and concentrations would confirm our initial findings. However, despite the small size of our cohort, significant differences following treatment with SIRT1 activators were detected, supporting the need to further validate on a larger cohort of individuals.

Based on the body of preclinical results reported here, further efforts should be spent to fully characterize the functions of ISIDE11 for its translation to clinical practice.

### Supplementary Information

Below is the link to the electronic supplementary material.

Supplementary file1 (DOCX 6875 KB)

## Data Availability

Data will be made available upon reasonable requests.
