# Supplementary material for: SIRT1 pharmacological activation rescues vascular dysfunction and prevents thrombosis in MTHFR deficiency
Source: Cell Mol Life Sci. 2022 Jul 11;79(8):410. doi: 10.1007/s00018-022-04429-5 (PMC9276577; doi:10.1007/s00018-022-04429-5)
Supplement: Supplementary file 1 — Supplementary file1 (DOCX 6875 KB) [file 18_2022_4429_MOESM1_ESM.docx]

**Supplemental FIGURES**

**SIRT1 pharmacological activation rescues vascular dysfunction and prevents thrombosis in MTHFR deficiency**

Albino Carrizzo^1,2^, Concetta Iside^3^, Angela Nebbioso^4^, Vincenzo Carafa^4^, Antonio Damato^2^, Sebastiano Sciarretta^2,5^, Giacomo Frati^2,5^, Flavio di Nonno^2^, Valentina Valenti^6^, Michele Ciccarelli^1^, Eleonora Venturini^2^, Mariarosaria Scioli^2^, Paola Di Pietro^1^, Tommaso Bucci^1^, Valentina Giudice^1^, Marianna Storto^1^, Bianca Serio^7^, Annibale Alessandro Puca^1,8^, Giuseppe Giugliano^9^, Valentina Trimarco^10^, Raffaele Izzo^9^, Bruno Trimarco^11^, Carmine Selleri^1,7^, Lucia Altucci^4,12^ and Carmine Vecchione^1,2^

^1^Department of Medicine, Surgery and Dentistry, “Scuola Medica Salernitana” University of Salerno, 84081, Baronissi, Italy; ^2^IRCCS Neuromed, Vascular Physiopathology Unit, 86077, Pozzilli, Italy; ^3^IRCCS Synlab SDN, Via E. Gianturco 113, Naples, 80143, Italy; ^4^Department of Precision Medicine, University of Campania "Luigi Vanvitelli", Vico L. De Crecchio, 80138, Naples, Italy; ^5^Department of Medico-Surgical Sciences and Biotechnologies, Sapienza University of Rome, 04100 Latina, Italy; ^6^Department of Cardiology, Santa Maria Goretti Hospital, 04100, Latina, Italy; ^7^University Hospital, San Giovanni di Dio e Ruggi D’Aragona, 84125, Salerno, Italy; ^8^IRCCS Multimedica, Ageing Unit, Via G. Fantoli 16/15, 20138, Milan, Italy; ^9^Department of Advanced Biomedical Sciences, "Federico II" University, Via Pansini 5, 80131 Naples, Italy; ^10^Department of Neuroscience, Reproductive Sciences and Dentistry, "Federico II" University, Via S. Pansini, 80131, Naples, Italy; ^11^International Translational Research and Medical Education (ITME) Consortium and Department of Advanced Biomedical Sciences, "Federico II" University, 80131, Naples, Italy; ^12^BIOGEM, Institute of Molecular Biology and Genetics, Via Camporeale, 83031, Ariano Irpino, Italy.

**Address correspondence to:** Carmine Vecchione, Department of Medicine, Surgery and Dentistry, “Scuola Medica Salernitana” University of Salerno, Via S. Allende, 84081 Baronissi (SA), Italy. Phone: +39089965069; Email: [cvecchione@unisa.it](mailto:cvecchione@unisa.it)

**Supplementary Figures**

**
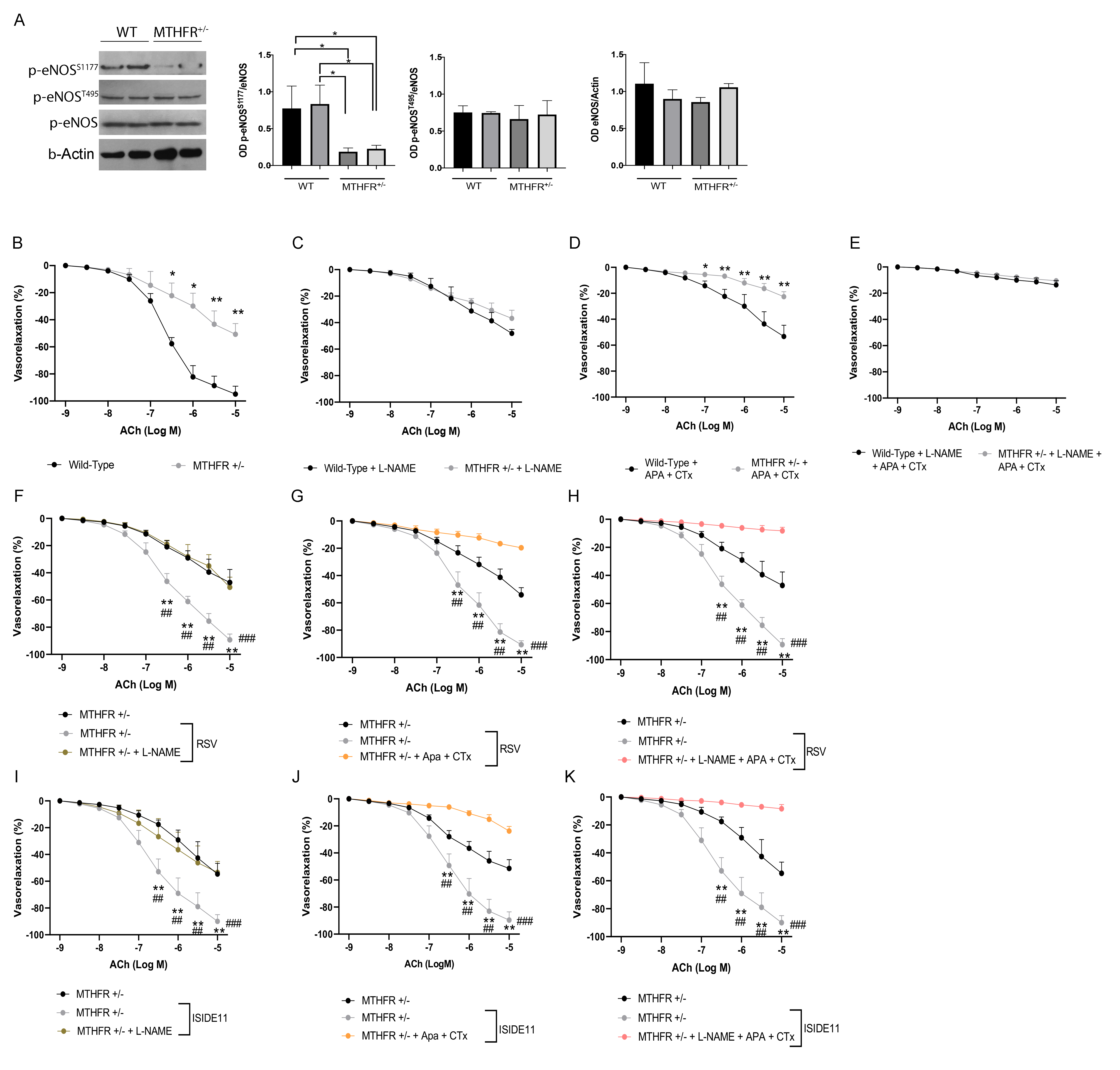
**

**Supp. Fig 1. (A)** Representative western blot analysis of mesenteric arteries obtained from wild-type and Mthfr+/- mice stimulated with acetylcholine and relative quantification of eNOS phosphorylation on serine 1177 and threonine 495. **(B-E)** Graphs show, from left to right, the ex vivo response of mouse mesenteric arteries obtained from wild-type and *Mthfr^+/-^* mice to endothelium-dependent vasorelaxant acetylcholine (ACh), in basal condition and in presence of L-NAME, or APA and CTx alone or in combination. (N=5 for each experiment). (**F-H**) Dose–response curves to acetylcholine (ACh) of mesenteric arteries obtained from *Mthfr^+/-^* mice in presence of eNOS inhibitor, L-NAME, or in presence of EDHF inhibitor (APA + CTx) or in presence of L-NAME plus APA plus CTx in combination after incubation for 1 hour with resveratrol. (**I-K**) Dose–response curves to acetylcholine (ACh) of mesenteric arteries obtained from *Mthfr^+/-^* mice in presence of eNOS inhibitor, L-NAME, or in presence of EDHF inhibitor (APA + CTx) or in presence of L-NAME plus APA plus CTx in combination after incubation for 1 hour with resveratrol. *, *p* < 0.05. **, *p* < 0.001; ***, *p* < 0.0001.

**Supp. Fig. 2** Identification and characterization of ISIDE11. (**A**) Flowchart of the screening procedure in HTS mode to identify new SIRT1 modulators. (**B**) SIRT1 *in vitro* assay at 10 μM concentration. **C**) Counter-screening at 10 μM concentration. Error bars indicate standard deviation (SD) of three biological replicates. Statistical analysis was performed using a Student’s t test. (**** p < 0.0001; ***, p < 0.0005; *, p < 0.0477). (**D**) AC_50_ evaluation based on dose-dependent enzymatic activity. Error bars indicate standard deviation (SD) of three biological replicates. Statistical analysis was performed using One-way ANOVA test. (****, p < 0.0001). **(E)** Western blot analyses of CETSA (top) and its analisys (down) by GraphPad Prism 6.0 Software. Statistical analysis was performed using a Student’s t test CONTROL vs ISIDE11: ***, p < 0.0002; CONTROL vs RSV: ns. Representative western blot of duplicates (N=2). **(F)** SIRT1–ISIDE11 interaction monitored by fluorescence spectroscopy. Tyrosine fluorescence emission as F0/F ratio was evaluated for both SIRT1 (blue line) and free tyrosine (red line) after addition of ISIDE11 at different concentrations (0, 0.5, 1, 5, 10, 20, 30, 50 µM). Working concentrations were 10 µM for both SIRT1 and free tyrosine. (**G**) Permeability analysis was perfomend in Caco-2 cells at 50 µM; ISIDE 11 (1 mg/ml) in 20% DMSO solution in 90% DMEM at 37°C. ISIDE11 was quantified at 375 nm. Error bars indicate standard deviation (SD) of three biological replicates. Statistical analysis was performed using a Student’s t test. ( **, p < 0.0025).

**
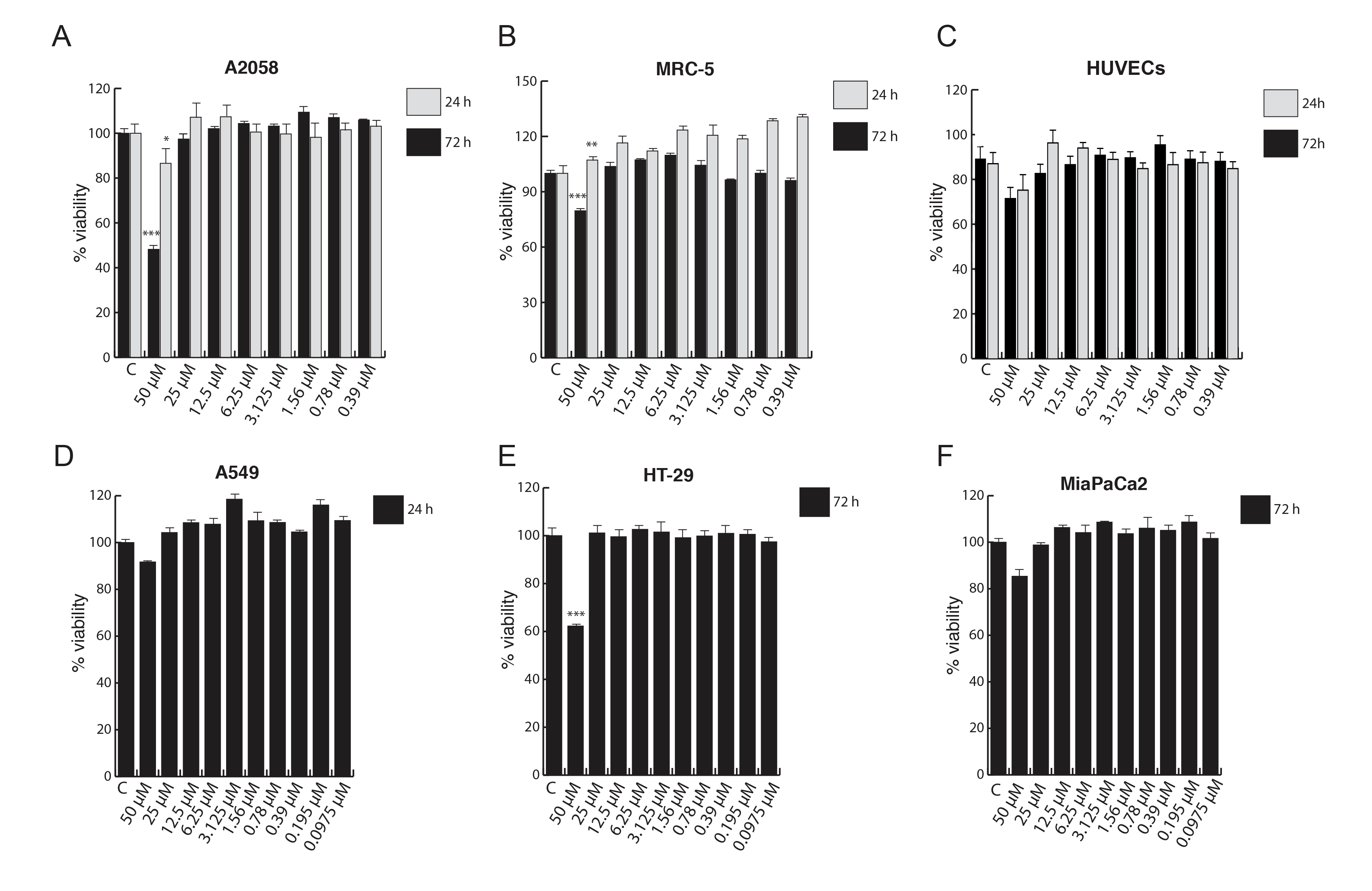
**

**Supp. Fig. 3** Effects of ISIDE11 on MTT assay. (**A**) A2058 cells were treated with ISIDE11 from 50 μM to 0.39 µM for 24 h and 72 h. *, p < 0.0149, ***, p < 0.0004 (**B**) MRC-5 cells were treated with ISIDE11 from 50 μM to 0.39 µM for 24 h and 72 h. **, p < 0.0014, ***, p < 0.0003 (**C**) Primary Umbilical Vein Endothelial Cells (HUVECs) were treated with ISIDE11 from 50 μM to 0.39 µM for 24 h and 72 h. (**D**) A549 cells were treated with ISIDE11 from 50 μM to 0.0975 µM for 24 h. (**E**) HT-29 cells were treated with ISIDE11 from 50 μM to 0.39 µM for 24 h. ***, p < 0.0009 (**F**) MiaPaCa cells were treated with ISIDE11 from 50 μM to 0.0975 µM for 72 h. Error bars indicate standard deviation (SD) of three biological replicates. Statistical analysis was performed using a Student’s t test.

**Supp. Fig. 4** ISIDE11 counteracts the effects of cell damage. Western blot performed in HepG2 cells for (**A**) p53 K382ac. Cells were pretreated for 6 h with etoposide and then treated with ISIDE11. ERK1/2 signal was used as loading control; (**B**) p53K382ac and p53. Cells were treated with etoposide, ISIDE11 and RSV alone and in combination as shown for 6 h. Actin was used as loading control; (**C**) Western blot for p53K379ac and H3K56ac. Cells were treated with etoposide and ISIDE11 alone and in combination for 6 h. ERK1/2 and Red Ponceau signals were used as loading controls. Quantification of the bands was performed by ImageJ software. Error bars indicate standard deviation (SD) of two biological replicates. Statistical analysis was performed using One-way ANOVA test. (****, p < 0.0001; ***, p < 0.0002; **, p < 0.0037; **, p < 0.0022; *, p < 0.0129). (**D** and **E**) SIRT1 and p53 mRNA fold expression was evaluated in HepG2 cells after treatment with ISIDE11 and EX-527 for 6 h. Error bars indicate standard deviation (SD) of two biological replicates. Statistical analysis was performed using a Student’s t test.(***, p < 0.0004).


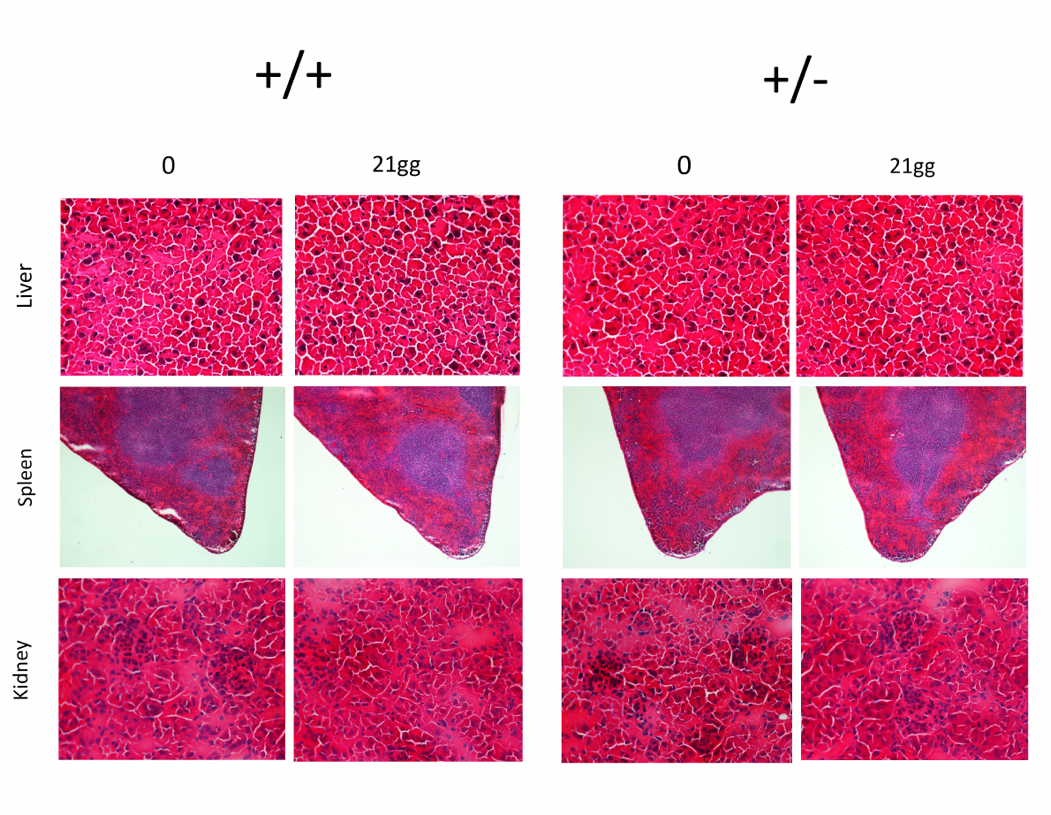
**Supp. Fig. 5.** Histopathological analysis of livers, spleens and kidneys of mice evaluated at baseline and after 21 days of treatment with ISIDE11. All sections were stained with hematoxylin/eosin. Kidney, liver and spleen images: 100x. (n=4/group).

**Supp. Table 1**

|  |  | ISIDE 11 |  |  |  | ISIDE 11 |  |
| --- | --- | --- | --- | --- | --- | --- | --- |
| Parameters | Wild-type | Wild-type | P value |  | MTHFR +/- | MTHFR +/- | P value |
| RBC (106 /mm3) | 8,45 ± 0,34 | 8,14 ±1,03 | *NS* |  | 8,53 ± 0,45 | 8,19 ± 0,68 | *NS* |
| Hematocrit (%) | 38,93 ± 1,85 | 38,83 ± 6,20 | *NS* |  | 39,6 ± 0,70 | 38,6 ± 1,80 | *NS* |
| Hemoglobin (g/dL) | 13,13 ± 0,85 | 13,0 ± 2,52 | *NS* |  | 13,53 ± 1,36 | 13,17 ± 2,04 | *NS* |
| MCV (fL) | 45,03 ± 1,29 | 46,7 ± 1,48 | *NS* |  | 45,63 ± 0,32 | 46,8 ± 2,35 | *NS* |
| MCH (pg) | 14,13 ± 0,99 | 13,83 ± 1,61 | *NS* |  | 13,8 ± 1,22 | 14,03 ± 2,02 | *NS* |
| MCHC (%) | 31,33 ± 3,02 | 30,2 ± 2,80 | *NS* |  | 30,9 ± 1,65 | 30,47 ± 2,25 | *NS* |
| WBC (103 /mm3) | 5,23 ± 1,24 | 5,18 ± 0,66 | *NS* |  | 5,83 ± 0,55 | 5,94 ± 0,8 | *NS* |
| Eosinophils (%) | 0,27 ± 0,06 | 0,28 ± 0,13 | *NS* |  | 0,24 ± 0,15 | 0,30 ± 0,14 | *NS* |
| Basophils (%) | 0,68 ± 0,04 | 0,63 ± 0,04 | *NS* |  | 0,60 ± 0,36 | 0,63 ± 0,23 | *NS* |
| Lymphocytes (%) | 80,13 ± 9,45 | 80,07 ± 9,57 | *NS* |  | 80,67 ± 7,22 | 80,8 ± 8,49 | *NS* |
| Monocytes (%) | 3,03 ± 0,68 | 3,60 ± 2,17 | *NS* |  | 3,23 ± 0,91 | 3,17 ± 1,01 | *NS* |
| Platelets (10^3^ /μL) | 806 ± 192 | 814 ± 125 | *NS* |  | 896 ± 179 | 835 ± 157 | *NS* |

**Supp. Table 1.** Haematological parameters in control or MTHFR +/- mice groups at baseline and after treated with ISIDE11 for 21 days. Red blood cells (RBC); mean corpuscular volume (MCV); mean corpuscular haemoglobin (MCH); mean corpuscular haemoglobin concentration (MCHC); white blood cells (WBC).
